# Innovative Advances in Droplet Microfluidics

**DOI:** 10.34133/research.0856

**Published:** 2025-08-27

**Authors:** Daohong Zhang, Wenkai Liu, Lang Feng, Yuming Feng, Yang Yu, Tinghai Cheng, Dong Han, Hengyu Li

**Affiliations:** ^1^ Beijing Institute of Nanoenergy and Nanosystems, Chinese Academy of Sciences, Beijing 101400, P. R. China.; ^2^ School of Nanoscience and Engineering, University of Chinese Academy of Sciences, Beijing 100049, P. R. China.; ^3^ State Key Laboratory of Fluid Power and Mechatronic Systems, Zhejiang University, Hangzhou 310027, P. R. China.

## Abstract

Droplet microfluidics is a rapidly evolving technology enabling precise control and manipulation of small-volume droplets, typically ranging from picoliters to nanoliters, offering important potential for biomedical applications. By generating highly uniform droplets with size variation below 5% and at high frequencies exceeding 10,000 droplets per second using techniques such as flow focusing, this approach facilitates high-throughput experimentation with minimal reagent consumption. These features make droplet microfluidics invaluable for single-cell analysis, drug screening, and disease diagnostics. Recent advancements in integrating droplet microfluidics with biological and clinical workflows have expanded possibilities for personalized medicine, early disease detection, and high-resolution cellular assays. This review provides an overview of recent progress in droplet microfluidics, focusing on key techniques for droplet generation, manipulation, and detection. It explores their applications in cutting-edge biomedical research, including single-cell analysis, 3-dimensional cell culture, drug development, and cancer research. Additionally, we discuss current challenges, such as improving reproducibility, scalability, and system integration, and outline promising future directions to fully realize the potential of droplet microfluidics in biomedicine.

## Introduction

Over the past 3 decades, microfluidics has garnered important attention and advanced rapidly. Droplet microfluidics, a key subset of this field, enables the manipulation of minute fluid volumes with exceptional precision and control [1,2]. In biomedicine, droplet microfluidics has transformed various applications by enabling high-throughput experiments, minimizing reagent consumption, and preventing cross-contamination, positioning it as a critical tool for single-cell analysis [3], drug discovery [4], disease modeling [5], and personalized medicine [6].

At the core of droplet microfluidics is the behavior of fluids at the microscale, characterized by low Reynolds numbers, which results in unique fluid dynamics and distinct behaviors. These properties underpin a wide range of biomedical applications [7,8]. By exploiting the interaction between immiscible fluids—typically a continuous and a dispersed phase—precisely controlled droplets are generated [9]. This encapsulation capability prevents cross-contamination, making droplet microfluidics ideal for high-throughput, parallelized assays, including multiplexed biomarker screening, drug testing, and cellular interaction studies [10,11].

The manipulation of droplets is a critical aspect of droplet microfluidics, particularly in biomedical applications, where precise control over droplet behavior is essential [12]. Various physical fields, including electrical, optical, thermal, acoustic, and magnetic fields, can be employed to manipulate droplets by altering fluid properties such as viscosity, surface tension, and flow rate [13,14]. These fields offer direct control over droplet size, frequency, and velocity, enabling precise manipulation of biological samples. Furthermore, by integrating microfluidic devices with emerging technologies like triboelectric nanogenerators (TENGs) [15], researchers can explore new avenues for droplet manipulation, offering enhanced control and expanded capabilities for biomedical applications [16,17].

The detection and analysis of droplets is another essential aspect of droplet microfluidics, especially in biomedicine. Droplets act as isolated microreactors, enabling studies on single cells, molecular interactions, and dynamic processes [18–20]. Advanced detection techniques enable real-time monitoring of droplet contents, allowing precise measurement of cell behavior, gene expression, and drug response [21–23].

**Fig. 1.**
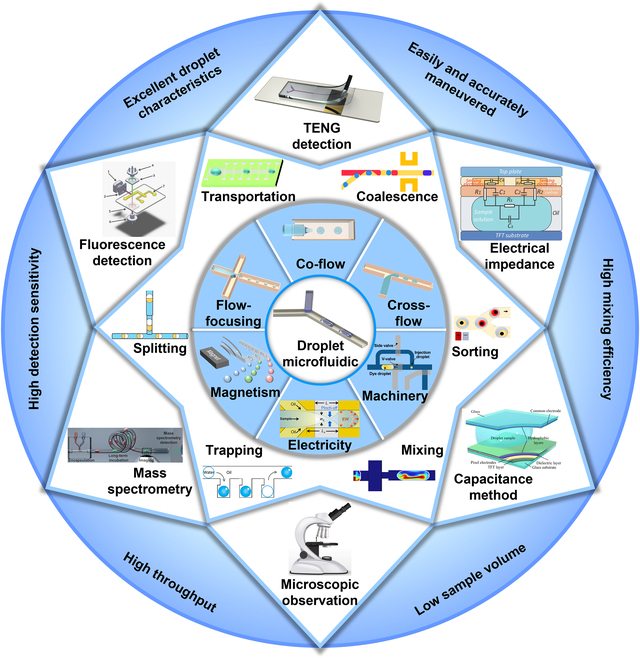
Overview of droplet microfluidic systems. Magnetism. Reprinted with permission from [157], Copyright 2023, American Chemical Society. Electricity. Reprinted with permission from [158], Copyright 2025, Royal Society of Chemistry. Machinery. Reprinted with permission from [159], Copyright 2023 Wiley-VCH. Mixing. Adapted from [54], Copyright 2024, Borzoki, licensed under CC BY 4.0. Trapping. Reprinted with permission from [160], Copyright 2017, American Chemical Society. Splitting. Reprinted with permission from [161], Copyright 2024, Elsevier. Transportation. Reprinted with permission from [162], Copyright 2017, Royal Society of Chemistry. Coalescence. Reprinted with permission from [163], Copyright 2020, Royal Society of Chemistry. Sorting. Reprinted with permission from [164], Copyright 2020, American Chemical Society. TENG detection. Reprinted with permission from [104], Copyright 2023, John Wiley and Sons. Electrical impedance. Adapted from [165], Copyright 2022, Jin et al., licensed under CC BY 3.0. Capacitance method. Adapted from [101], Copyright 2024, Jiang et al., licensed under CC BY 4.0. Mass spectrometry. Adapted from [85], Copyright 2024, Wink et al., licensed under CC BY 4.0. Fluorescence detection. Reprinted with permission from [166], Copyright 2013, Royal Society of Chemistry.

This review provides a comprehensive overview of droplet microfluidics in biomedicine, focusing on its applications in drug discovery, disease diagnostics, and personalized medicine. It examines recent advancements in droplet generation, manipulation, and detection, comparing methods and their respective advantages and limitations. The review is structured into 4 sections: (a) droplet generation techniques, (b) droplet manipulation strategies, (c) droplet detection methods, and (d) biomedical applications (Fig. 1). Through this analysis, we aim to highlight the transformative potential of droplet microfluidics in advancing both biomedical research and clinical applications.

Unlike many existing reviews, this manuscript provides a comprehensive and systematic overview covering droplet generation, manipulation, and detection. We place special emphasis on emerging noncontact droplet manipulation methods—including electric, magnetic, and acoustic approaches—and novel self-powered droplet detection technologies based on TENGs. Furthermore, this review highlights the integration of these advanced techniques with key biomedical applications such as single-cell analysis, 3-dimensional (3D) cell culture, drug development, and disease diagnostics. By bridging fundamental techniques and cutting-edge applications, our work offers an interdisciplinary perspective and provides insight into future research directions, including multifunctional integration, self-powering, and intelligent systems.

## Droplet Generation

### Passive methods

Passive droplet generation methods primarily rely on adjusting the microfluidic channel structure to control droplet formation. The 3 primary microchannel configurations are cross-flow, co-flow, and flow-focusing.

#### Cross-flow

**Fig. 2.**
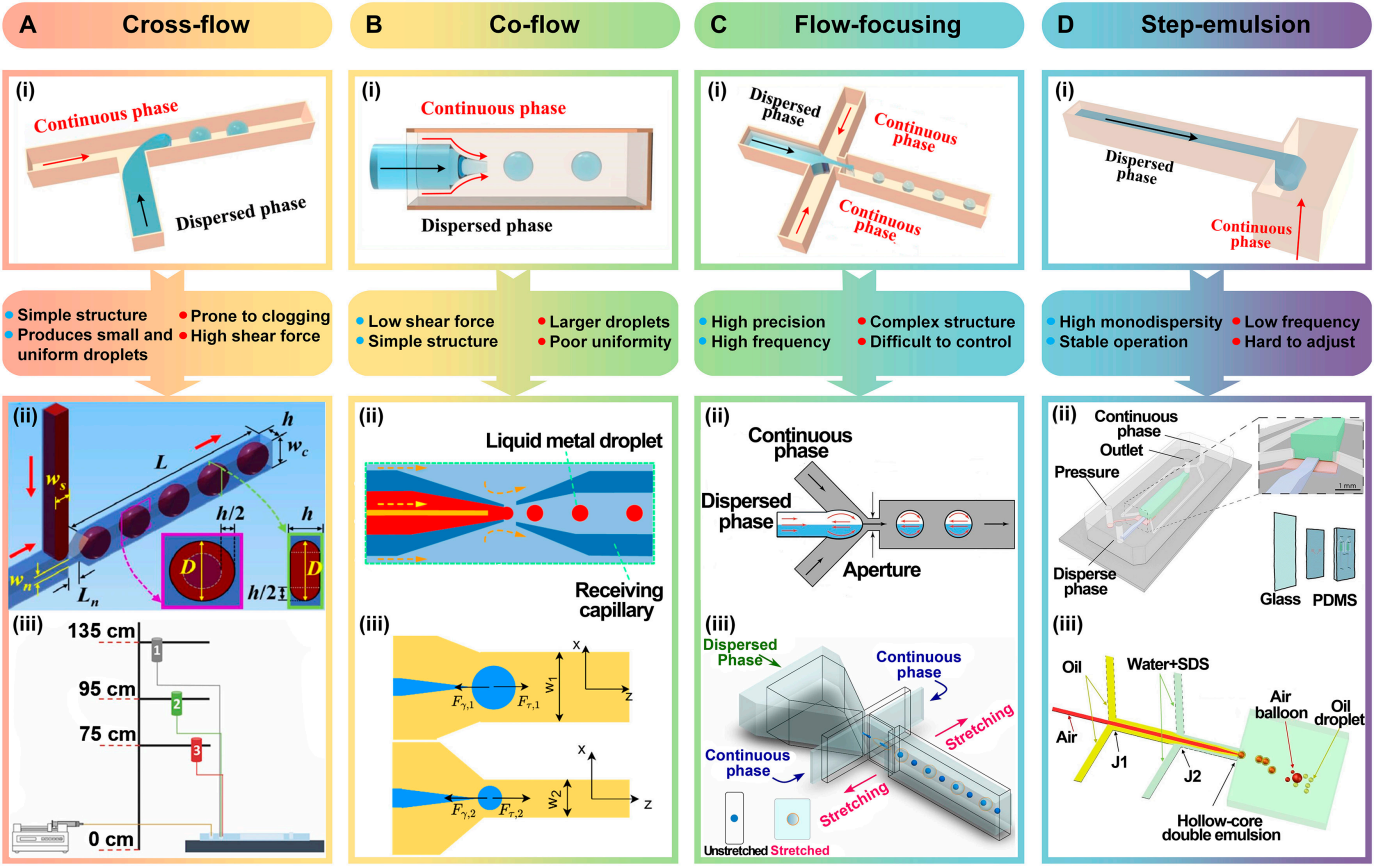
Passive methods. (A) Cross-flow. (i) T-junction device. (ii) T-junction with necked structure. Reprinted with permission from [24], Copyright 2020, John Wiley and Sons. (iii) Gravity-driven system for high-flux droplet generation. Adapted from [25], Copyright 2024, Bajgiran et al., licensed under CC BY 4.0. (B) Co-flow. (i) Co-flow device. (ii) Co-flow microfluidic device for liquid metal droplet formation. Adapted from [26], Copyright 2024, Hu et al., licensed under CC BY 4.0. (iii) Co-flow microfluidic device with flexible walls. Adapted from [27], Copyright 2024, Yazdanparast et al., licensed under CC BY 4.0. (C) Flow-focusing. (i) Flow-focusing device. (ii) Asymmetric flow-focusing device. Adapted from [28], Copyright 2024, Belousov et al., licensed under CC BY 4.0. (iii) Stretchable flow-focusing device. Adapted from [29], Copyright 2024, Roshan et al., licensed under CC BY 4.0. (D) Step-emulsion. (i) Step-emulsion device. (ii) Tunable parallelized step emulsification. Adapted from [31], Copyright 2024, Nalin et al., licensed under CC BY 4.0. (iii) Gas-assisted microfluidic step emulsification. Adapted from [32], Copyright 2024, Huang et al., licensed under CC BY 4.0.

Cross-flow refers to the generation of droplets occurring when the continuous phase and the dispersed phase intersect at an angle, with the continuous phase truncating the dispersed phase under the influence of pressure and shear. The T-junction [Fig. 2A(i)] is the most common configuration for cross-flow droplet generation. This method is favored for its simplicity, ease of fabrication, and low cost, making it a popular choice in microfluidics research. For instance, Pang et al. [24] examined the geometry of the T-junction and proposed a novel configuration featuring a neck at the junction akin to a flow-focusing channel, aiming to achieve smaller droplets, as depicted in Fig. 2A(ii). Furthermore, Bajgiran et al. [25] proposed a droplet generation method driven by gravity, which simultaneously generates droplets at 3 T-junctions. By designing reservoirs at different heights, the liquids flow to the T-junctions at varying velocities under the influence of gravity, thereby producing droplets of different sizes or frequencies, as shown in Fig. 2A(iii).

The cross-flow structure offers high efficiency, uniformity, and flexible control. However, it also presents challenges, including large chip size, high pressure loss, and the generation of relatively large droplets.

#### Co-flow

Co-flow is achieved through coaxial microchannels, where the dispersed phase flows through the inner channel and the continuous phase flows through the outer channel. Droplet formation is primarily driven by the shear forces exerted by the continuous phase on the dispersed phase [Fig. 2B(i)]. Co-flow chips are mainly made of glass capillaries, and thus are particularly suitable for the production of uniform emulsions and dual emulsion droplets, and provide enhanced solvent compatibility. Based on the phase-field model, Hu et al. [26] proposed an axisymmetric numerical model for 2-phase flow and systematically investigated the effects of continuous phase viscosity, flow rate, and interfacial tension on the formation of liquid metal droplets in a co-flow microfluidic device [Fig. 2B(ii)]. Yazdanparast et al. [27] developed a co-flow microfluidic device with flexible walls, which allows for the adjustment of channel width as needed, thereby enabling control over droplet size [Fig. 2B(iii)].

Co-flow offers precise control over droplet size by adjusting the 2-phase flow rates, providing a broad range of size control. However, at high flow rates, droplet merging may occur if shear forces are insufficient to separate the droplets, or when excessive flow velocity leads to droplet collisions, which can limit the formation rate.

#### Flow focusing

In flow-focusing chips, the continuous phase flows from both sides of the dispersed phase, promoting droplet formation through shear-induced constriction. The narrowing of the channel enables stable and uniform droplet formation [Fig. 2C(i)]. Flow focusing is particularly effective for producing smaller, monodisperse droplets.

Belousov et al. [28] proposed a novel asymmetric flow-focusing droplet generation device. This device utilizes asymmetric vortices in the droplet formation region to enhance mixing during droplet generation and achieves efficient mixing within a specific volume range, as illustrated in Fig. 2C(ii). Roshan et al. [29] proposed a stretchable microfluidic device that dynamically alters channel shapes in real time to generate variable-sized droplets, as shown in Fig. 2C(iii).

Flow focusing can generate highly uniform droplets with precise control over droplet size due to its unique structure, making it suitable for applications requiring monodisperse droplets. Additionally, its multiple inlet channels facilitate the generation of multiphase droplets. However, the complexity of its design and manufacturing, along with higher costs, remain important drawbacks.

#### Step emulsion

In step emulsification chips, droplets are formed as the dispersed phase flows through a narrow microchannel and encounters an abrupt expansion, typically referred to as a “step”. The sudden widening of the channel geometry causes a rapid reduction in confinement, leading to droplet pinch-off driven by interfacial tension [Fig. 2D(i)]. Unlike shear-dominated mechanisms, step emulsification relies primarily on geometric and surface energy imbalances, making droplet formation less sensitive to flow rate variations. This method is particularly effective for generating highly monodisperse droplets under low capillary number conditions. George et al. [30] proposed a step emulsification method based on 2 successive pipetting steps, which enables the rapid generation of a large number of droplets from a minimal volume of highly viscous samples. Nalin et al. [31] developed tuna-step, a step emulsification-based microfluidic module with a pressure-actuated membrane that enables on-demand tuning of nozzle size and droplet volume, allowing the fabrication of 3D hydrogel structures with compositional gradients and adjustable porosity, as shown in Fig. 2D(ii). Huang et al. [32] proposed a gas-assisted co-flow step emulsification device, in which air serves as the innermost phase and induces oil droplet formation through gas diffusion, as shown in Fig. 2D(iii). Ota and Hashimoto [33] achieved droplet generation with a coefficient of variation below 2% by optimizing the triangular nozzle design in a step emulsification microchip.

Step emulsification is advantageous for its simplicity, scalability, and exceptional droplet uniformity. It is well suited for digital assays and applications that require precise volume control, such as digital polymerase chain reaction (PCR) and single-cell encapsulation. However, compared to shear-based methods, its droplet generation rate is typically lower, and its application may be limited under high-flow conditions. Table 1 provides a comparative overview of step emulsification, cross-flow, co-flow, and flow-focusing geometries.

**Table 1.** Passive droplet generation methods

Peculiarity	Cross-flow [24,25]	Co-flow [26,27]	Flow-focusing [28,29]	Step-emulsion [31,32]
Droplet diameter	5–180 μm	20–62.8 μm	5–65 μm	38.2–110.3
Generation frequency	2 Hz	1,300–1,500 Hz	850 Hz	33 Hz
Advantages	Simple structure, produces small and uniform droplets	Low shear force, simple structure, low cost	High precision, wide applicability, high frequency	Simple structure, high monodispersity
Disadvantages	Prone to clogging, high shear force	Larger droplets, poor uniformity	Complex structure, difficult to control	Low frequency, droplet size hard to adjust
Applications	Chemical synthesis	Biomedical	Drug delivery	Single-cell analysis

### Droplet generation by active methods

Active droplet generation employs external forces to drive and control droplet formation, which can be categorized into electrical, thermal, magnetic, and mechanical forces.

#### Electrical

The electrical control method induces microdroplet generation by applying voltage to the fluid in the microchannel. Traditional approaches to electrical control include utilizing an electric field and electrowetting.

**Fig. 3.**
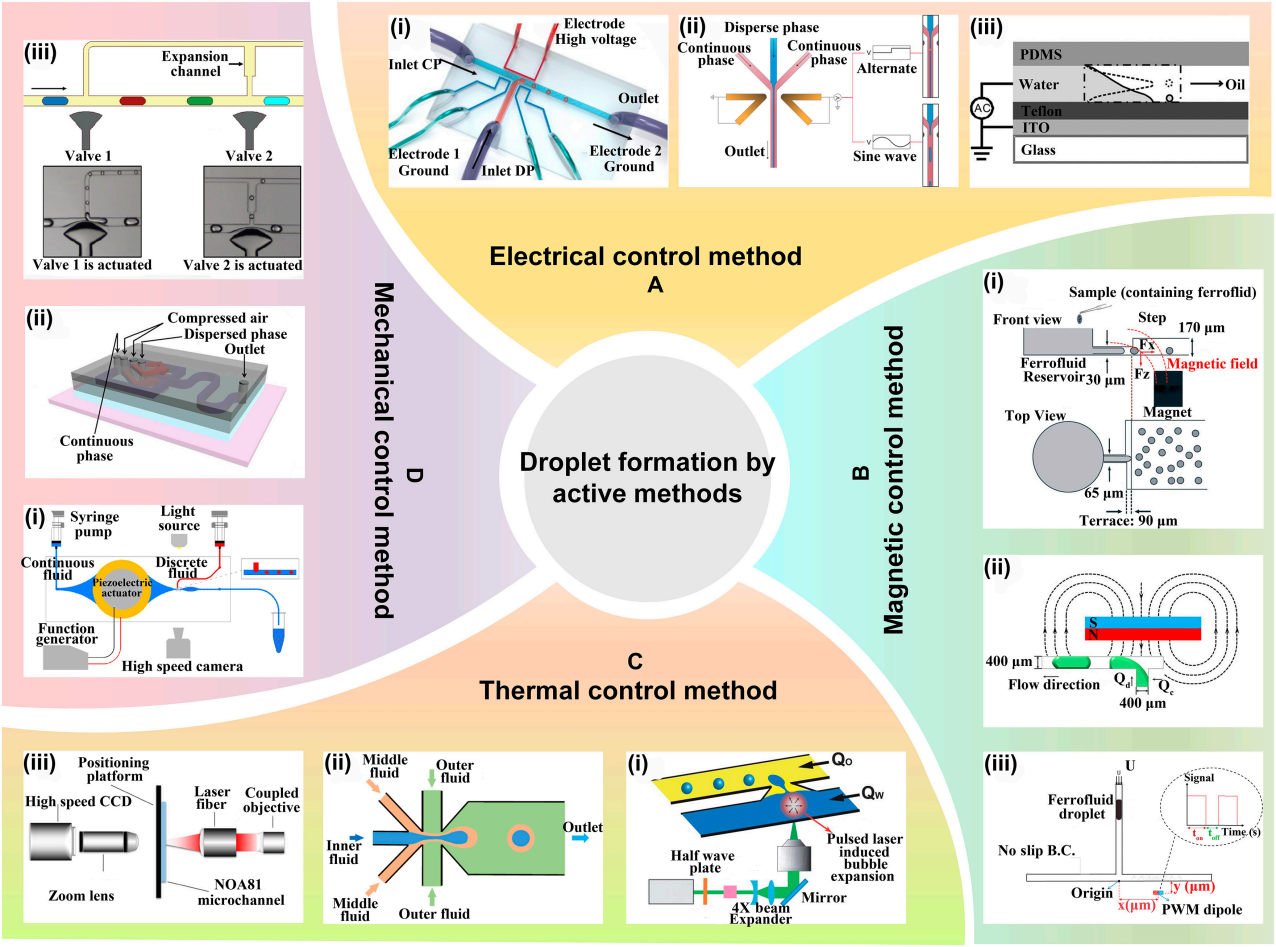
Active methods. (A) Electrical control method. (i) Flow-focusing device with integrated electrowetting. Reprinted with permission from [34], Copyright 2022, American Institute of Physics. (ii) T-junction microchannel with electric field. Reprinted with permission from [35], Copyright 2019, American Chemical Society. (iii) Droplet generation via DC, square, and sinusoidal wave. Reprinted with permission from [36], Copyright 2008, American Institute of Physics. (B) Magnetic control method. (i) Step emulsifying device via external magnetic field. Reprinted with permission from [37], Copyright 2016, Royal Society of Chemistry. (ii) Droplet generation by nonuniform magnetic field. Reprinted with permission from [38], Copyright 2018, Elsevier. (iii) Effect of pulse modulated magnetic field on droplet generation. Reprinted with permission from [39], Copyright 2021, Royal Society of Chemistry. (C) Thermal control method. (i) Laser pulse-induced droplet generation. Reprinted with permission from [40], Copyright 2014, Royal Society of Chemistry. (ii) Thermoelectric cooler-induced droplet generation. Reprinted with permission from [41], Copyright 2020, Elsevier. (iii) Focused infrared laser controls droplet generation. Adapted from [42], Copyright 2018, Wang et al., licensed under CC BY 4.0. (D) Mechanical control method. (i) Droplet generation regulated by a piezoelectric resonator. Reprinted with permission from [43], Copyright 2022 Elsevier. (ii) Microfluidic chip with pneumatic diaphragm valve. Reprinted with permission from [44], Copyright 2016, Springer Nature. (iii) On-demand droplet using double T-junctions with microvalves. Reprinted with permission from [45], Copyright 2020, American Chemical Society.

The electric field method is realized by applying either a DC or AC signal to the electrode. Liu et al. [34] employed the 3D lattice Boltzmann method and the leakage medium model to examine the effects of electric capillary number, flow ratio, and viscosity ratio on droplet formation in T-junction microchannels under electric field, as depicted in Fig. 3A(i). Yin et al. [35] discussed droplet generation under DC electric field, square wave, and sine wave, as illustrated in Fig. 3A(ii). In a sinusoidal AC field, high voltage and frequency leads to a higher rupture rate of the dispersed phase and a reduction in droplet size.

Electrowetting involves applying voltage to alter the wetting characteristics of the fluid–solid interface. This changes the contact angle between the fluid and the surface, causing the fluid front to extend and retract, which leads to droplet formation upon rupture. Gu et al. [36] successfully integrated electrowetting into a flow-focusing microfluidic device, achieving precise control of droplet size in the range of 5 to 50 μm, as depicted in Fig. 3A(iii).

The electric control method offers a simple structure, easy integration of electrodes, straightforward drive control, and favorable biocompatibility. However, the system’s design and manufacturing can be costly, and factors such as the electrical conductivity and surface tension of the liquid must be finely tuned to optimize droplet generation efficiency.

#### Magnetic

Noncontact magnetic field control methods are primarily based on ferrofluids. These fluids exhibit nonmagnetic properties in their static state but become magnetized when exposed to a magnetic field.

Kahkeshani and Carlo [37] utilized an external magnetic field to achieve rapid emulsification of ferrofluid solutions, generating different droplets by adjusting fluid viscosity, interfacial tension, and magnetic field strength, as depicted in Fig. 3B(i). Zhang et al. [38] established a nonuniform magnetic field using a permanent magnet to explore the droplet generation characteristics at T-junctions under magnetic field influence. They observed 3 droplet generation patterns—slug flow, slug flow drip transition, and drip flow, as illustrated in Fig. 3B(ii). Bijarchi et al. [39] employed the finite element volume method and the volume of fluid (VOF) 2-phase model to investigate the impact of pulse-width-modulated magnetic fields on T-junction droplet generation, as shown in Fig. 3B(iii). Their study examined the effects of magnetic bond number, duty cycle, magnetic field frequency, and other factors on droplet division.

Magnetic microdroplets prepared using ferrofluids offer advantages such as controllable motion, straightforward operation, good biocompatibility, and excellent dispersion, making them extensively utilized in biomedicine.

#### Thermal

The thermal control method generates droplets by altering fundamental fluid properties, such as viscosity and interfacial tension, through temperature changes. Common thermal control techniques include local laser heating and internal resistance heating.

Park et al. [40] proposed a novel mechanism for droplet generation propelled and driven by pulsed laser action. This approach involved the induction of cavitation bubbles within water via laser pulses, disrupting the stable oil–water interface and facilitating water ingress into the oil channel, thereby facilitating droplet generation, as depicted in Fig. 3C(i). Wu et al. [41] devised a microfluidic chip regulation system predicated based on a custom-designed thermoelectric cooler, as shown in Fig. 3C(ii), to produce double-emulsion drops. The observed trends indicated that as the temperature increased, the frequency of droplet generation increased, while both inner and outer diameters decreased. Wang et al. [42] employed focused infrared laser irradiation to control droplet generation and examined the associated characteristics under varying flow rates, laser power settings, and spot positions, as depicted in Fig. 3C(iii).

The thermal control method features a relatively simple design structure and allows for accurate droplet generation control. However, achieving precise temperature regulation remains challenging. Moreover, the thermal properties of the fluid, such as boiling point and viscosity, may limit the effectiveness and scalability of the method.

#### Mechanical

The mechanical method typically achieves droplet generation by utilizing hydraulic and pneumatic components, and piezoelectric elements. Zhang and Xia [43] utilized a piezoelectric disk actuator for active droplet generation, enabling precise control over droplet generation frequency and volume by manipulating the input signal of the piezoelectric actuator, as illustrated in Fig. 3D(i). Cai et al. [44] devised a controllable nozzle utilizing a pneumatic film valve, which was integrated into a multi-layer polydimethylsiloxane (PDMS) chip for rapid and precise generation and adjustment of microdroplets with varying sizes, as demonstrated in Fig. 3D(ii). Agnihotri et al. [45] presented a droplet microfluidic device that incorporated a double T-junction and microvalve. By regulating the microvalve, they achieved on-demand droplet generation and splitting, allowing for the production of droplets with varying volumes, as depicted in Fig. 3D(iii).

Mechanical control technology is relatively mature, providing high reliability and stability. It effectively handles various types of liquids, including those with high viscosity and complex properties. However, challenges such as the size and wear of mechanical components considerably limit its application. Additionally, mechanical control tends to be less flexible compared to other methods, especially in terms of dynamic droplet size or frequency adjustment. Table 2 summarizes the active droplet generation.

**Table 2.** Summary of active methods

Peculiarity	Electric [34–36]	Magnetic [37–39]	Thermal [40–42]	Machine [43–45]
Droplet diameter	10–500 μm	50–500 μm	50–500 μm	200–2,000 μm
Generation frequency	50 Hz–10 kHz	30 Hz–10 kHz	1–1,000 Hz	1–100 Hz
Advantages	Fast response, noncontact	Noncontact, good flexibility	Suitable for high-viscosity liquids	Easy to adjust, precise control
Disadvantages	Complex structure, bubble-prone	Requires magnetic liquid	Poor applicability, slow response	Complex structure, contamination
Applications	Microreactors, nanomaterial synthesis	Microbial culture, chemical synthesis	High-viscosity fluid processing	Cell culture, microsphere preparation

### Conclusion

Droplet generation lies at the heart of droplet microfluidic systems, fundamentally shaping the droplet’s size, uniformity, frequency, and its compatibility with downstream processes such as manipulation, detection, and application-specific integration. Both passive and active droplet generation methods offer distinct advantages and limitations, and their selection should be guided by the specific functional requirements of the intended application.

Passive methods, including cross-flow, co-flow, flow focusing, and step emulsification, rely on channel geometry and fluidic parameters to create droplets. Cross-flow and flow-focusing configurations, which operate at high shear rates, provide stable and rapid droplet production suitable for high-throughput applications and integration with optical or electrical detection techniques. However, they may be sensitive to flow rate fluctuations, affecting droplet volume control. Co-flow systems excel in chemical resistance and are particularly favorable for generating double emulsions, making them ideal for drug delivery and bio-encapsulation. Step emulsification, characterized by excellent monodispersity and low flow rate requirements, is highly compatible with sensitive biological samples and critical applications like single-cell analysis and digital PCR, although it is less suitable for real-time droplet manipulation due to lower throughput.

Active methods, on the other hand, leverage external forces—electrical, magnetic, thermal, or mechanical—to achieve programmable, on-demand droplet production with high precision. Electrical control methods, such as electric fields and electrowetting, enable high-frequency, size-tunable droplet formation for digital microfluidics and lab-on-a-chip platforms, although fluid conductivity and interfacial properties must be carefully managed. Magnetic control, using ferrofluids, offers noncontact and biocompatible droplet manipulation for biomedical applications, albeit limited by fluid type. Thermal control adjusts viscosity and interfacial tension via localized heating, allowing fine control of droplet characteristics for temperature-sensitive or multi-phase systems, but heat-induced instability may hinder use in biological contexts. Mechanical actuation, including piezoelectric, pneumatic, and hydraulic systems, provides robust and versatile droplet generation across diverse fluid types, but may require larger device footprints and offer limited miniaturization flexibility.

In summary, the choice between passive and active droplet generation methods should consider key factors such as throughput, droplet uniformity, compatibility with sensitive samples, integration with downstream functions (e.g., merging, sorting, detection), and the physical constraints of the microfluidic system.

## Droplet Manipulation

Precise droplet manipulation is fundamental to droplet microfluidic applications in biochemistry and plays a crucial role in advancing subsequent research endeavors. Typically, passive droplet manipulation can be achieved by adjusting the channel structure and fluid properties, while active manipulation requires external excitation methods, such as electric, magnetic, and acoustic forces.

### Droplet manipulation in channel

In conventional droplet microfluidics, droplet manipulation is usually realized within a closed channel of a microfluidic chip, including droplet sorting, droplet mixing, droplet splitting, and droplet trapping.

#### Sorting

**Fig. 4.**
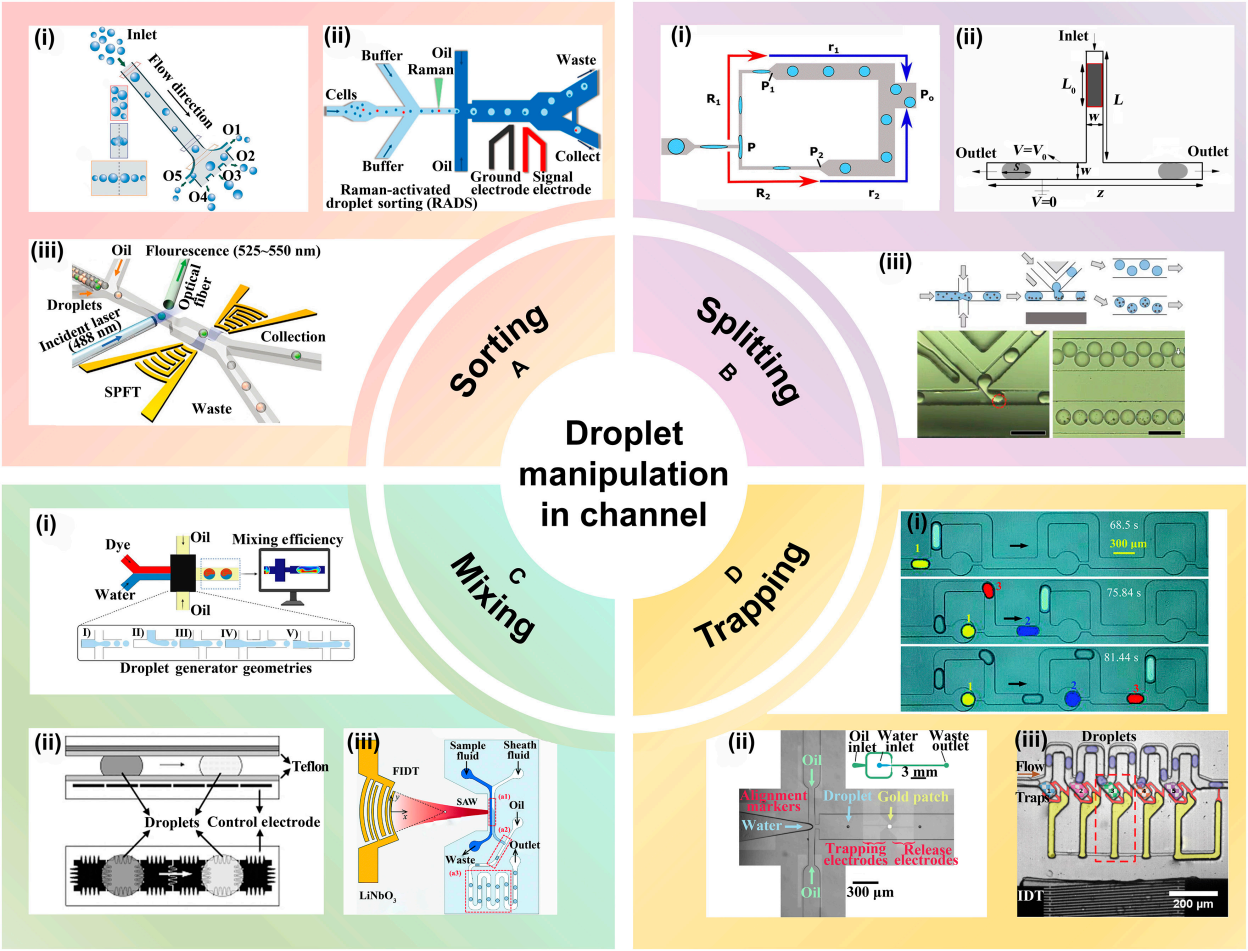
Droplet manipulation inside chip. (A) Sorting. (i) Inertia-based sorting of hydrogel droplets. Reprinted with permission from [46], Copyright 2018, Royal Society of Chemistry. (ii) Raman-activated droplet sorting system. Reprinted with permission from [47], Copyright 2017, American Chemical Society. (iii) Acoustic chip with SPFT for droplet sorting. Reprinted with permission from [49], Copyright 2021, John Wiley and Sons. (B) Splitting. (i) Micro-elastofluidics for tunable droplet splitting. Reprinted with permission from [51], Copyright 2025, Royal Society of Chemistry. (ii) Droplet splitting under asymmetric electric field. Adapted from [52], Copyright 2022, Fallah et al., licensed under CC BY 4.0. (iii) Magnetic bead droplet splitting in K-channels. Reprinted with permission from [53], Copyright 2017, American Chemical Society. (C) Mixing. (i) Droplet mixing via crossed T-junctions. Adapted from [54], Copyright 2024, Borzoki, licensed under CC BY 4.0. (ii) Electrowetting-driven high-speed droplet oscillation. Reprinted with permission from [55], Copyright 2003, Royal Society of Chemistry. (iii) Acoustofluidic droplet mixing method. Reprinted with permission from [56], Copyright 2020, Royal Society of Chemistry. (D) Trapping. (i) Bubble-assisted droplet trapping device. Adapted from [57], Copyright 2018, Zhang et al., licensed under CC BY 4.0. (ii) Droplet trapping and release via 2 pairs of electrowetting electrodes. Adapted from [58], Copyright 2016, Pit et al., licensed under CC BY 4.0. (iii) Surface sound waves inducing bubbles for droplet trapping. Adapted from [59], Copyright 2017, Rambach et al., licensed under CC BY-NC 3.0.

Droplet sorting involves transporting droplets to various downstream channels based on specific conditions, making it a crucial step for subsequent droplet analysis. Factors such as droplet size, gravity, and viscosity serve as the criteria for sorting, facilitating distinct droplet movements within designated channels. For instance, Li et al. [46] introduced an inertial microfluidic device where hydrogel droplets of varying sizes exhibit different equilibrium positions in the microtube due to shear gradient lift and wall effect lift. Larger hydrogel droplets tend to align closer to the centerline of the channel compared to smaller ones, as illustrated in Fig. 4A(i).

Active droplet sorting is achieved by applying external stimuli such as electric and acoustic fields, with dielectrophoresis (DEP) being the most prevalent method. The principle of DEP involves placing droplets in an asymmetric electric field, resulting in the generation of dielectrophoretic forces due to dielectric polarization. For instance, Wang et al. [47] developed a Raman-activated droplet separation microfluidic system for label-free, high-throughput screening of living cell functions. Droplets were analyzed using Raman microspectral analysis and separated via DEP, as depicted in Fig. 4A(ii).

TENGs, introduced by Z. L. Wang’s group in 2012, are gaining prominence in droplet manipulation. By utilizing TENG as a drive source, the high output voltage generated through friction electrification is applied to electrodes, enabling droplet manipulation via electrowetting-on-dielectric (EWOD) and DEP. Zhou et al. [48] combined TENG with microfluidic technology, using the alternating electric field generated by TENG to achieve AC electroosmosis and induced electroosmosis. This method effectively mixed 2 fluids at a frequency of 10 Hz and demonstrated precise control over particle behavior, including aggregation, deflection, and separation.

Acoustic droplet sequencing has garnered considerable attention due to its excellent biocompatibility. Zhong et al. [49] introduced a droplet sorting acoustic fluidic chip utilizing a single-phase focusing sensor (SPFT). By detecting droplet fluorescence signals via optical fiber, pulse acoustic signals were generated to trigger SPFT, enabling droplet sorting, as illustrated in Fig. 4A(iii). Zhong et al. [50] proposed a droplet sorting system based on electrical impedance and acoustic sorting technologies. By removing multicellular and empty droplets, the system selectively sorts single-cell droplets, achieving a sorting efficiency of 90.3% and a throughput of 200 Hz, showing potential for improving sorting purity and accelerating single-cell screening.

Other droplet separation methods, including magnetic field-induced separation and pneumatic valve utilization, offer alternative approaches for droplet sorting, further expanding the toolkit for droplet-based applications.

#### Splitting

Droplet splitting involves dividing a single droplet into 2 or more smaller droplets, enabling high-throughput droplet generation and optimizing the use of small-volume samples. The simplest method for droplet splitting is the Y-junction, which splits a single droplet into 2 separate droplets. Roshan et al. [51] achieved tunable droplet splitting by externally stretching a T-junction structure, which enabled real-time adjustment of channel wall dimensions and precise control over the volume and ratio of the daughter droplets, as depicted in Fig. 4B(i).

Active droplet splitting approaches encompass electrical, magnetic, and mechanical methodologies. For instance, Fallah and Fattahi [52] conducted a numerical investigation into droplet splitting at a T-junction under an asymmetric electric field. By applying an electric field to one branch of the microchannel, droplets of various sizes could be generated, with precise control over the number of droplets splits achievable through manipulation of electric field intensity, as illustrated in Fig. 4B(ii). Magnetic droplet splitting involves the entrapment of magnetic beads within the droplet for sample concentration and washing. Doonan devised a K-shaped junction that, when subjected to a magnetic field, facilitates the division of a single droplet into 2 while trapping magnetic beads within one of them [53], as shown in Fig. 4B(iii).

#### Mixing

Droplet mixing is the process of combining multiple droplets, which is crucial for facilitating reagent reactions within them. Due to the typically laminar flow of fluids in microfluidic channels, mixing between different phases occurs primarily through molecular diffusion, resulting in relatively slow mixing rates. To overcome this challenge, various channel structures have been developed for passive droplet mixing. Kheirkhah Barzoki [54] introduced 3 innovative cross-T structures that integrate cross and T-junctions to enhance droplet mixing efficiency, decrease droplet diameter, and mitigate the risk of contamination due to droplet-wall contact, as depicted in Fig. 4C(i).

Active droplet mixing techniques encompass electrowetting and sonic-induced droplet mixing. For instance, Paik et al. [55] employs electrowetting to manipulate droplet mixing by modulating the interfacial tension of droplets via applied voltage, treating the droplets as virtual mixing chambers, and acoustically transmitting them through an array of electrodes for mixing, as illustrated in Fig. 4C(ii). Park et al. [56] proposed an acoustofluidic method that couples acoustic waves with fluid flow. By generating controlled acoustically induced microvortices, this approach enables efficient mixing of aqueous solutions, as depicted in Fig. 4C(iii).

#### Trapping

Droplet trapping refers to the process of positioning droplets in specific locations within a microfluidic device for further manipulation. Passive droplet trapping is achieved through the design of specialized channel structures. For example, Zhang et al. [57] proposed a bubble-assisted droplet trapping device. By introducing air pillars in the microchannel to guide the formation of bubbles and utilizing the relatively high hydrodynamic resistance of the bubbles, the device enables efficient droplet trapping, as illustrated in Fig. 4D(i).

There are 2 main types of active droplet trapping mechanisms. One involves direct droplet trapping through external force application, as demonstrated by Pit et al. [58]. They utilized 2 pairs of carefully arranged electrically wetted electrodes: The first pair temporarily traps the droplet, while the second pair pulls the droplet away from the initially trapping electrodes to effectuate release, depicted in Fig. 4D(ii). The other type involves transporting droplets into traps via external force. For instance, Rambach et al. [59] utilized LiNbO_3_ crystals to generate surface acoustic waves. When these waves acted on the middle portion of the 2 traps, bubbles were formed. Upon deactivation of the surface acoustic waves, the bubbles dissipated, subjecting the droplets to considerable Laplace pressure, thereby guiding them into the traps, as illustrated in Fig. 4D(iii).

#### Others

In response to the growing complexity of droplet-based microfluidic systems, researchers have developed a range of advanced droplet manipulation techniques beyond the conventional operations of sorting, splitting, mixing, and trapping. For instance, Cui et al. [60] proposed a microfluidic injector for droplet encoding. The device features a circular electrode and concentric channel design, forming multiple injection ports. By altering the fluorescent components within the droplets, binary and gradient encoding of droplet sequences was successfully achieved. In another study, Fang et al. [61] introduced a multi-component droplet coalescence device controlled by an AC electric field. By adjusting the electric field parameters, rapid droplet coalescence under various flow conditions was realized within milliseconds. These innovative approaches have been widely adopted in complex workflows such as multi-step biochemical reactions, high-throughput screening, and single-cell analysis, where precise and versatile droplet control is essential.

### Digital microfluidic droplet manipulation

Digital microfluidics is a technique used to process discrete droplets on a substrate, usually in a working environment on the surface of the substrate, which can be precisely controlled to realize operations such as droplet generation, transport, splitting, and merging.

#### Transport

**Fig. 5.**
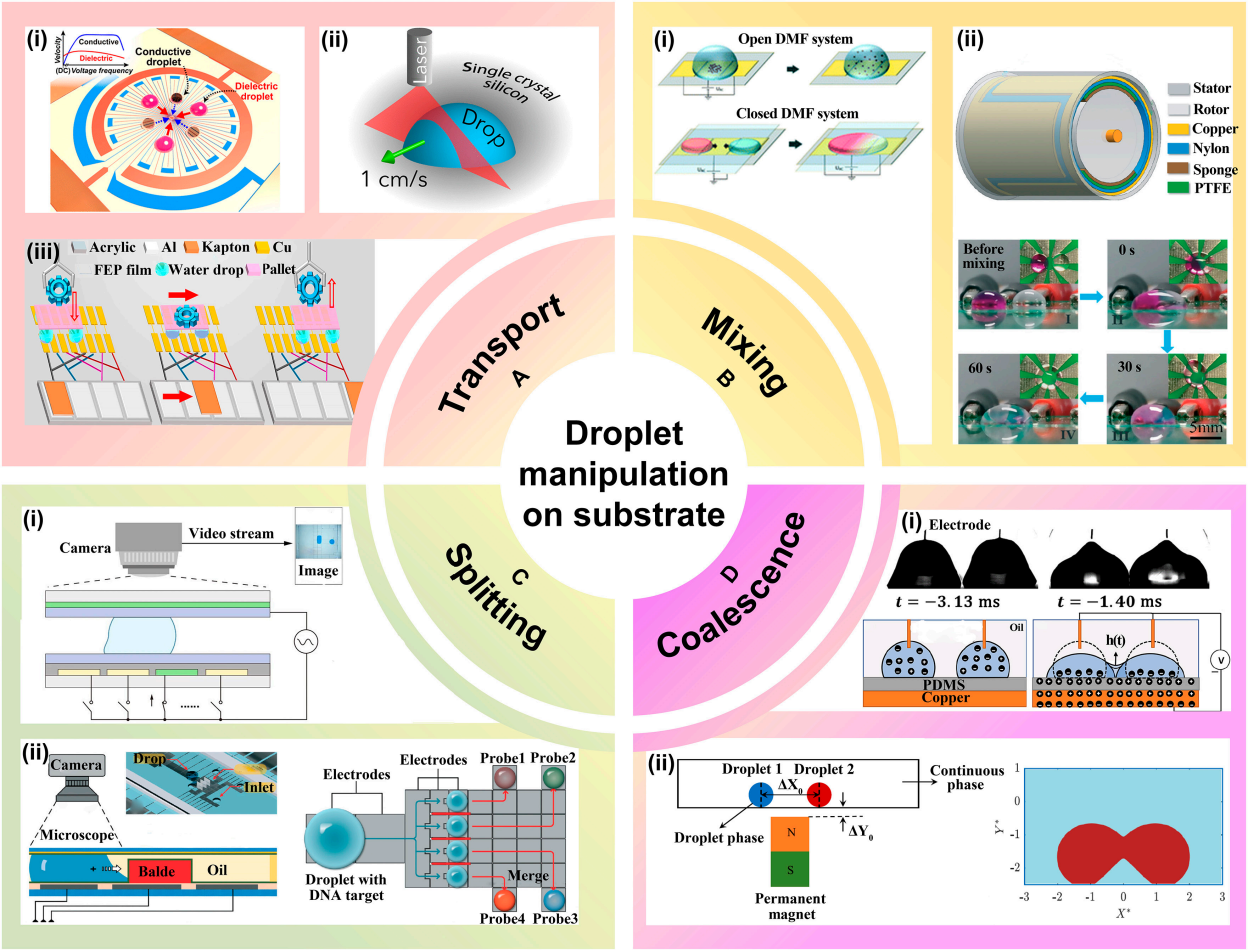
Droplet manipulation on substrate. (A) Transport. (i) Electric field-driven directional droplet transport platform. Reprinted with permission from [62], Copyright 2024, American Chemical Society. (ii) Droplet transport via OEW. Reprinted with permission from [63], Copyright 2018, American Chemical Society. (iii) Self-powered droplet transport system combining TENG and EWOD. Reprinted with permission from [64], Copyright 2018, American Chemical Society. (B) Mixing. (i) Electrohydrodynamic technique for rapid mixing. Reprinted with permission from [66] , Copyright 2017, Royal Society of Chemistry. (ii) TENG drives DEP for droplet mixing. Reprinted with permission from [67], Copyright 2021, Royal Society of Chemistry. (C) Splitting. (i) AI-assisted digital microfluidic. Adapted from [68], Copyright 2024, Guo et al., licensed under CC BY 4.0. (ii) Highly accurate droplet splitting using 3D blade structures. Adapted from [69], Copyright 2017, Dong et al., licensed under CC BY-NC 3.0. (D) Coalescence. (i) Droplet coalescence of detached droplet under electrowetting induction. Reprinted with permission from [70], Copyright 2020, American Chemical Society. (ii) Permanent magnets induce droplet coalescence. Reprinted with permission from [71], Copyright 2021, American Chemical Society.

Droplet transport, defined as the directional and continuous movement of liquid on a plane, plays a crucial role in applications such as biomedical detection and microchemical reactions. Among the various methods, EWOD stands out as the most common approach. EWOD involves the application of an electric potential at a specific position between one side of the droplet and the substrate, thereby altering the droplet’s surface tension and inducing movement on the hydrophobic surface. Building upon this foundation, Wu et al. [62] proposed an electric field-driven directional droplet transport platform. By leveraging the dielectric constant differences among droplets, the system enables coordinated transport of multiple droplets, flexible combinations of various droplet types, and multifunctional droplet transport modes, as depicted in Fig. 5A(i).

However, conventional wetting methods encounter limitations, particularly in scenarios where the droplet transport path is complex. This complexity necessitates an increased number of electrodes for wetting, leading to wiring challenges and overall structural intricacy. Optoelectric wetting, which leverages light patterns to control wetting effects, emerges as a promising solution to this bottleneck. Palma and Deegan [63] utilized the optoelectrowetting (OEW) effect to drive droplets on a substrate using localized light beams. This approach effectively eliminates the need for electrode arrays in conventional EWOD systems, thereby reducing the complexity associated with their fabrication and control, as illustrated in Fig. 5A(ii).

Another innovative solution comes from TENGs, which have demonstrated excellent performance in droplet transport and offer substantial versatility in microfluidic applications. For instance, Nie et al. [64] integrated electric wetting techniques with TENG to develop a self-powered microfluidic transport system. This system uses a micro-vehicle device propelled by 4 droplets to transport a pallet that can carry tiny objects. The droplets, which rest on a hydrophobic surface, are manipulated by the Coulomb forces generated by the TENG-driven output voltage acting on electrodes beneath the surface, as illustrated in Fig. 5A(iii).

#### Mixing

In digital microfluidics, electrowetting remains the conventional approach for droplet mixing. This method utilizes AC-driven planar electrodes to induce mixing through electrowetting oscillations [65]. Dong et al. [66] proposed a digital microfluidic platform capable of achieving isothermal hydrodynamic flow-enhanced droplet mixing. This is accomplished using a single electrode to induce droplet boundary oscillations driven by low-frequency AC, thereby facilitating accelerated droplet mixing, as illustrated in Fig. 5B(i). Additionally, TENG-driven DEP represents another promising method for droplet mixing. Yu et al. [67] introduced a self-powered droplet manipulation system integrating 3 technologies: TENG, EWOD, and DEP. In this system, TENG provides power to EWOD and DEP, with EWOD facilitating long-distance droplet transport and DEP responsible for droplet mixing, as depicted in Fig. 5B(ii).

#### Splitting

Existing methods for droplet splitting on digital microfluidic typically utilize external forces, such as electricity and magnetism, for microdroplet manipulation. For instance, Guo et al. [68] developed an artificial intelligence (AI)-assisted digital microfluidic framework based on droplet morphology. The system automatically identifies the state and interactions of droplets and implements feedback control via electrical actuation, as depicted in Fig. 5C(i). However, a drawback of this method is the uneven splitting of droplets. Addressing this issue, Dong et al. [69] introduced a 3D micro-blade structure, integrating a set of electrodes with 3D micro-blades positioned appropriately according to the desired splitting ratio. Sequential charging of the electrodes enables simultaneous droplet splitting as needed, as illustrated in Fig. 5C(ii).

#### Coalescence

Droplet coalescence involves the coalescence of 2 or more droplets into a singular entity following degeneration upon contact and rupture of the interface separating them. This process constitutes a pivotal stage in facilitating reactions between disparate component droplets.

Quintero et al. [70] investigated 3 distinct scenarios of droplet coalescence induced by the electro-wetting method. The first scenario involves single-electrode electro-aggregation, where electrodes propel droplets and attract others for merging. However, the merging process in this method lacks regularity. In the second scenario, 2-electrode electro-aggregation, 2 electrodes drive respective droplets until contact is achieved. However, electrostatic repulsion arises between the droplets at this juncture. The final scenario also utilizes 2 electric levels, but droplets are detached from the electrodes, allowing electric field manipulation for merging without electrostatic repulsion, as depicted in Fig. 5D(i). Another approach is to use a magnetic field for droplet coalescence. Hassan et al. [71] explored droplet merging on hydrophobic surfaces under the influence of a permanent magnetic field, where a nonuniform magnetic field brings droplets into proximity, leading to eventual merging, as shown in Fig. 5D(ii).

### Conclusion

Precise droplet manipulation is essential for enabling a wide range of applications in droplet microfluidics, including high-throughput screening, biochemical reactions, and single-cell analysis. Both channel-based and digital microfluidic approaches offer diverse strategies for droplet operations such as sorting, splitting, mixing, trapping, and coalescence. Passive techniques leverage microchannel geometry and fluid dynamics, while active methods—based on electric, acoustic, magnetic, and triboelectric forces—provide enhanced control and responsiveness. The integration of emerging technologies such as TENGs, optoelectric wetting, and 3D microstructures has expanded the capabilities and flexibility of droplet manipulation platforms. Moving forward, the trend toward miniaturization, automation, and multifunctional integration will continue to drive innovation in droplet control strategies, further advancing their utility in complex biological, chemical, and diagnostic workflows.

## Droplet Detection Method

Droplet detection is a critical component of droplet microfluidics, enabling the monitoring of droplet characteristics such as shape, internal constituents, and count, as well as other inherent properties. It provide versatile solutions for droplet analysis, facilitating applications across different fields of research and industry.

### Optical detection

Optical detection is widely used in droplet microfluidic systems to analyze the physical and chemical properties of droplets by measuring the interaction of light with the droplets. Common optical detection methods can be classified into 3 categories: transmission and reflection-based detection, scattering and imaging detection, and fluorescence and spectral detection.

#### Transmission and reflection detection

Transmission and reflection-based detection are commonly used in droplet microfluidic systems, primarily for analyzing the physical and chemical properties of droplets. Transmission detection quantifies internal properties of droplets, such as solute concentration and particle distribution, by measuring light transmission changes. Reflection detection evaluates surface properties—such as surface tension, roughness, and morphology—by analyzing variations in reflected light intensity. This method is particularly useful for monitoring surface characteristics and morphological changes of droplets.

**Fig. 6.**
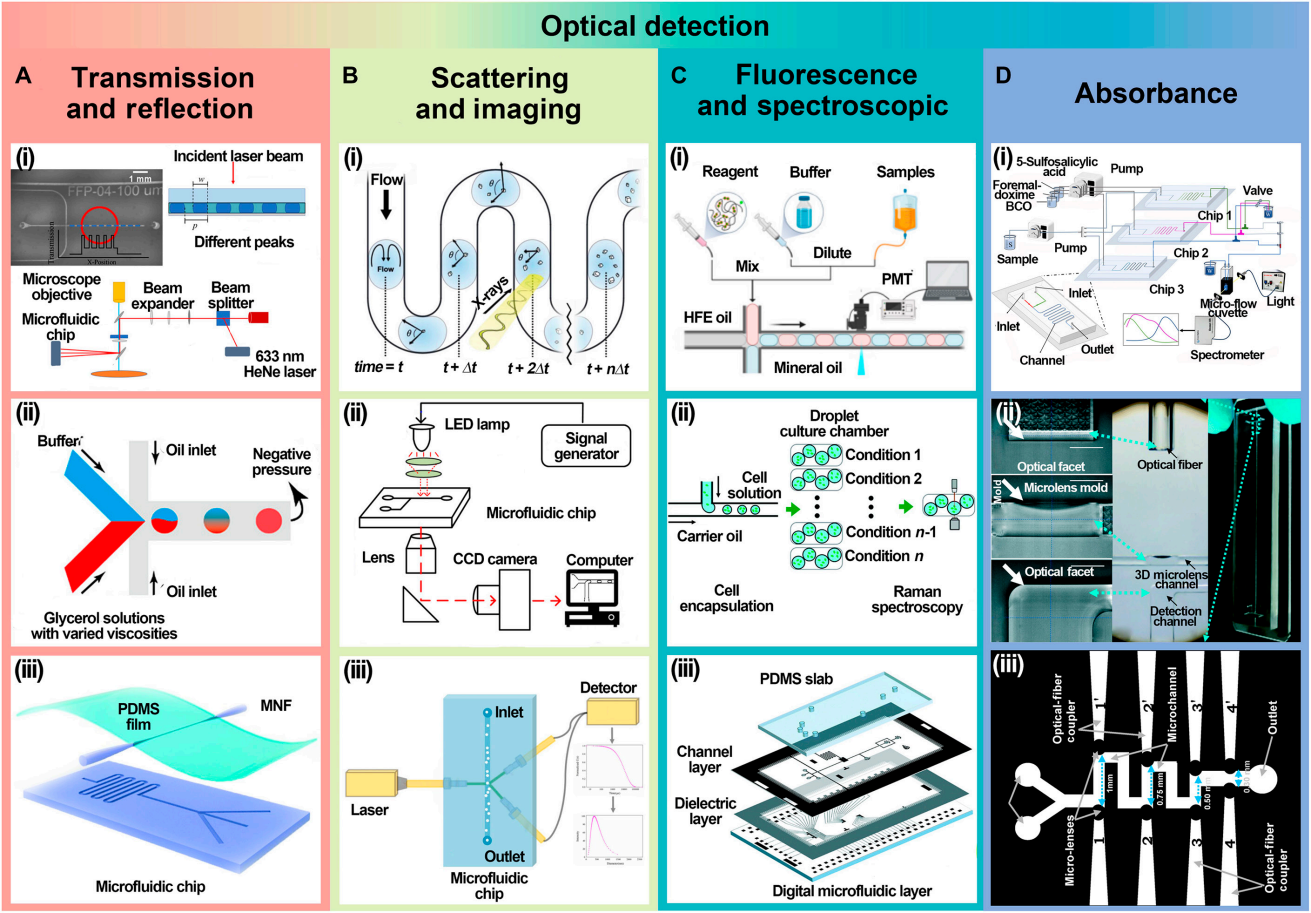
Optical detection. (A) Transmission and reflection detection. (i) Label-free droplet analysis using dynamic phase grating. Reprinted with permission from [72], Copyright 2023, American Chemical Society. (ii) Instant blood coagulation detection. Reprinted with permission from [73], Copyright 2022, American Chemical Society. (iii) Flow sensing based on optical micro/nanofiber embedded in soft membranes. Reprinted with permission from [74], Copyright 2020, Royal Society of Chemistry. (B) Scattering and imaging detection. (i) Droplet microfluidic combined with XRD analysis. Reprinted with permission from [75], Copyright 2019, John Wiley and Sons. (ii) Visualizing and regulating droplet behavior in microfluidic systems. Adapted from [76], Copyright 2019, Gao et al., licensed under CC BY 4.0. (iii) Dual-angle fiber-optic DLS system combined. Reprinted with permission from [77], Copyright 2022, Elsevier. (C) Fluorescence and spectroscopic detection. (i) Real-time monitoring of pancreatic α-amylase in patients. Reprinted with permission from [78], Copyright 2024, Elsevier. (ii) Raman spectroscopy-compatible droplet culturing and analysis system. Reprinted with permission from [79], Copyright 2017, Royal Society of Chemistry. (iii) Integrated droplet digital microfluidic system. Reprinted with permission from [80], Copyright 2019, Royal Society of Chemistry. (D) Absorbance detection. (i) Flow injection analysis system. Reprinted with permission from [81], Copyright 2025, Elsevier. (ii) 3D microlens-integrated microfluidic chip. Adapted from [82], Copyright 2018, Tang et al., licensed under CC BY-NC 3.0. (iii) Microfluidic device for detecting the concentration of heavy metal ions. Reprinted with permission from [83], Copyright 2022, Elsevier.

Mesyngier and Bailey [72] introduced a label-free droplet analysis method utilizing dynamic phase gratings, as shown in Fig. 6A(i), which leverages phase interference effects from both transmitted and reflected light, enabling precise measurement of droplet properties, including size, shape, and concentration, without the need for external markers. Additionally, Chen et al. [73] proposed a droplet microfluidic method for instant blood clotting detection, as shown in Fig. 6A(ii). This method dynamically monitors changes in blood viscosity to evaluate the clotting process. Furthermore, Zhang et al. [74] developed a novel flow sensing technology utilizing optical micro/nanofibers embedded in soft membranes [Fig. 6A(iii)], integrating optical fibers and membranes to enable multifunctional flow detection.

In summary, transmission and reflection detection methods play a crucial role in droplet microfluidic systems. By accurately measuring the interaction between light and droplets, these methods provide quantitative analysis of the physical and chemical properties of droplets. However, droplet size and composition can affect detection accuracy. This requires high sample homogeneity. Moreover, multi-parameter detection increases data processing complexity, requiring advanced algorithms for accurate results.

#### Scattering and imaging detection

Scattering and imaging detection are primarily used to analyze particle distribution, morphological changes, and other physical properties of droplets. Scattering detection analyzes the intensity and angle of scattered light resulting from the interaction between light and particles within the droplet. This method provides information on particle size, distribution, and concentration and is commonly used for dynamic monitoring and analysis of particulate matter. Imaging detection, on the other hand, captures high-resolution images of the droplet’s morphology, motion, and surface features. It enables real-time monitoring of changes in droplet shape, flow behavior, and collision responses, making it suitable for morphological analysis, microstructural characterization, and real-time observation.

Levenstein et al. [75] combined droplet microfluidics with x-ray diffraction (XRD) to identify effective nucleating agents for calcium carbonate crystal formation [Fig. 6B(i)]. The platform allows precise control over reactant volumes and reaction conditions, while XRD analyzes the crystal structure of the products. Additionally, Gao et al. [76] proposed a strategy for visualizing and regulating droplet behavior within a microfluidic system [Fig. 6B(ii)], using optical imaging and control techniques to enable real-time observation and precise manipulation of droplet movement and merging. Furthermore, Chen et al. [77] introduced a dual-angle optical fiber dynamic light scattering (DLS) system integrated with a microfluidic chip, as shown in Fig. 6B(iii). The system uses optical fiber technology and DLS principles, leveraging multiple scattering angles to improve the accuracy and sensitivity of particle size measurements.

Integration of optical imaging and scattering techniques has substantially enhanced the performance of droplet microfluidic systems, improving accuracy and sensitivity. These advancements not only optimize droplet control within microfluidic systems but also enable precise measurement of microparticle properties in liquids.

#### Fluorescence and spectroscopy

Fluorescence detection excites fluorescent markers or naturally fluorescent molecules in droplets and measures the emitted light’s intensity and wavelength, enabling sensitive detection of target molecules. It is especially effective for the quantitative analysis of low-concentration samples or biomolecules, offering high selectivity and sensitivity. Spectroscopy analyzes the absorption, reflection, or scattering of light by droplet components to obtain spectral information, which is widely used for both qualitative and quantitative analysis.

Zhao et al. [78] developed a portable droplet microfluidic platform for real-time monitoring of pancreatic α-amylase in postoperative patients [Fig. 6C(i)]. This method tracks α-amylase activity by detecting signal changes as the enzyme reacts with specific substrates, offering a sensitive, real-time assessment of enzyme concentration in the body. Additionally, Kim et al. [79] introduced a droplet microfluidic platform compatible with Raman spectroscopy for lipidomics research on chips [Fig. 6C(ii)]. The platform integrates microfluidic technology with Raman spectroscopy, allowing cell or biomolecule culture and real-time analysis within the microfluidic chip. Furthermore, Ahmadi et al. [80] proposed an integrated droplet digital microfluidic system that can generate, mix, incubate, and sort droplets on demand [Fig. 6C(iii)]. This system leverages digital microfluidic technology for precise control of droplet generation and manipulation, enhancing operational efficiency in biological analysis, chemical reactions, and cell screening.

In conclusion, fluorescence and spectroscopy detection are powerful tools for molecular analysis and low-concentration substance detection in droplet microfluidic systems. These technologies enable sensitive detection of target molecules in droplets, allowing fast, noninvasive, real-time monitoring, particularly for biomedical, environmental, and chemical analysis applications.

#### Absorbance detection

Absorbance detection is a commonly used optical detection method in droplet microfluidics. Its basic principle is to infer the concentration or composition of a sample based on the amount of light it absorbs at a specific wavelength. When droplets pass through the detection region, light emitted from a source travels through the droplet, where part of it is absorbed by the substances within. The remaining light is captured by a detector. According to the Beer–Lambert law, the absorbance is proportional to the concentration of the analyte in the solution, thereby enabling quantitative detection of target substances within the droplet.

Li et al. [81] developed a flow injection analysis system based on a multiplexed microfluidic chip, utilizing a fiber optic spectrometer for real-time absorbance signal detection [Fig. 6D(i)]. This system enabled the simultaneous detection of iron, copper, and manganese ions in samples, demonstrating the practical application of absorbance detection in multi-ion analysis scenarios. Tang et al. [82] employed 2-photon polymerization to develop a 3D microlens-integrated microfluidic chip with a large detection channel [Fig. 6D(ii)]. The integration of 3D microlenses into the microchannel considerably improved the signal-to-noise ratio and optical coupling efficiency in absorbance detection, enabling precise detection of tartrazine samples. Mishra et al. [83] integrated absorbance detection with microfluidic chip technology and proposed a microfluidic device for detecting the concentration of water-soluble heavy metal ions [Fig. 6D(iii)].

In summary, absorbance detection offers a straightforward and label-free method for quantitative analysis in droplet microfluidics. Its compatibility with microfluidic platforms and ability to provide real-time, continuous measurements make it a valuable tool in various analytical applications.

### Mass spectrometry

By combining microfluidic technology with mass spectrometry (MS), real-time detection of trace substances in droplets is achievable, making it suitable for fields such as biomedical research, environmental monitoring, and chemical analysis. This technology enables precise separation and capture of target substances and allows efficient qualitative and quantitative analysis, greatly enhancing the analytical capabilities of droplet microfluidic systems for complex samples. Common MS techniques include electrospray ionization MS (ESI-MS), matrix-assisted laser desorption/ionization MS (MALDI-MS), and liquid chromatography–MS (LC-MS) coupling.

#### Electrospray ionization MS

**Fig. 7.**
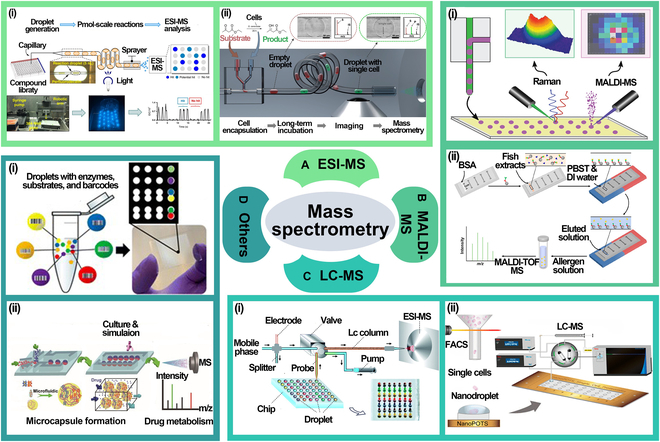
Mass spectrometry. (A) ESI-MS. (i) High-throughput platform combining ESI-MS and droplet microfluidics for photochemical reactions. Adapted from [84], Copyright 2020, Sun et al., licensed under CC BY 4.0. (ii) Microfluidic chip cell biocatalytic transformation analysis using ESI-MS. Adapted from [85], Copyright 2022, Wink et al., licensed under CC BY 4.0. (B) MALDI-MS. (i) Combines droplet microfluidic and MALDI-MS for GABA analysis. Adapted from [87], Copyright 2021, Bell et al., licensed under CC BY 4.0. (ii) Uses droplet microfluidics with MALDI-TOF MS to evaluate fish allergens cross-reactivity. Reprinted with permission from [88], Copyright 2022, American Chemical Society. (C) LC-MS. (i) Combines LC-MS and droplet array for label-free enzyme inhibition analysis. Reprinted with permission from [89] , Copyright 2014, Royal Society of Chemistry. (ii) Nanodroplet processing platform. Reprinted with permission from [90], Copyright 2020, American Chemical Society. (D) Other MS methods. (i) Direct injection of single cells for analysis using inductively coupled plasma quadrupole MS (ICP-qMS). Reprinted with permission from [91], Copyright 2020, American Chemical Society. (ii) Drop-NIMS platform. Reprinted with permission from [92], Copyright 2024, Royal Society of Chemistry.

ESI-MS is a highly sensitive, high-resolution technique widely used for analyzing liquid samples, especially complex biomolecules, metabolites, and pharmaceutical compounds. ESI works by generating charged droplets from the liquid sample via an electric field, leading to the production of gas-phase ions suitable for mass analysis without extensive fragmentation. Sun et al. [84] proposed a high-throughput platform based on droplet microfluidics for exploring photochemical reactions [Fig. 7A(i)]. This platform generates small droplets to rapidly screen and optimize reaction conditions. ESI-MS is used for online analysis of reaction products, leveraging its high sensitivity to detect product composition and changes under varying conditions, enabling efficient evaluation of reaction efficiency and selectivity. Wink et al. [85] combined droplet microfluidic technology with MS for quantitative analysis of the biocatalytic conversion process of single microbial cells [Fig. 7A(ii)]. Using a customized system, individual microbial cells are encapsulated in droplets for precise monitoring of the biocatalytic reaction, with MS providing high-sensitivity quantitative analysis of the reaction products. Payne et al. [86] demonstrated a method for droplet sorting based on signal intensity from ESI-MS, which enables screening of cell variants grown in 20-nl droplets at a rate of 0.5 samples per second.

The combination of ESI-MS and droplet microfluidics has made considerable progress in reaction screening, metabolite detection, and biocatalysis research, particularly excelling in high-throughput analysis and trace sample detection. However, this integration still faces several challenges. For instance, droplet size, composition, and uniformity can affect electrospray stability, impacting MS sensitivity and accuracy. ESI-MS also faces low ionization efficiency with high-salt or high-concentration solutions, which can reduce data reliability. Addressing these issues requires optimizing droplet control and sample pretreatment to ensure high-quality analysis in complex conditions. Despite these challenges, the combination of ESI-MS and droplet microfluidics holds broad application prospects.

#### Matrix-assisted laser desorption/ionization MS

MALDI is a soft ionization technique that uses a laser to desorb and ionize molecules embedded in a matrix, allowing large biomolecules like proteins and metabolites to be analyzed without fragmentation. By mixing reaction products in droplets with MALDI, MALDI-MS provides quick molecular information and enables single-droplet or single-cell level analysis. Bell et al. [87] applied droplet microfluidics combined with MALDI-MS for γ-aminobutyric acid (GABA) analysis [Fig. 7B(i)]. By optimizing the oil phase, they improved GABA analysis sensitivity and accuracy, offering valuable guidance for droplet microfluidics in biochemical and metabolomics research. Zhao et al. [88] combined droplet microfluidic chips with MALDI time-of-flight MS (MALDI-TOF MS) to assess fish allergen cross-reactivity [Fig. 7B(ii)]. They loaded protein extracts from different fish species onto the chip and analyzed them with MALDI-TOF MS, revealing allergen similarities and differences.

While the combination of MALDI-MS and droplet microfluidics offers considerable advantages, it also faces challenges. The choice of matrix and the concentration of reaction products can affect sensitivity and accuracy. Droplet size and uniformity also impact analysis precision, particularly in single-molecule or single-cell studies. MALDI-MS requires higher sample concentrations, and signal intensity may be insufficient for low-concentration samples.

#### Liquid chromatography–MS

The liquid sample is passed through a column packed with a stationary phase, where different molecules separate based on their interactions with the column material. MS provides molecular identification, mass analysis, and structural confirmation by ionizing chemical species and measuring their mass-to-charge ratios. The combination of LC and MS, coupled with the high-throughput capabilities of droplet microfluidics—which allows manipulation of tiny fluid volumes in discrete droplets—enables rapid, precise analysis of trace substances in complex systems.

Wang et al. [89] introduced a novel method combining LC-MS detection technology with microfluidic droplet arrays for label-free enzyme inhibition analysis [Fig. 7C(i)]. Williams et al. [90] developed a nanodroplet processing platform that enables fully automated sample injection from nanopores into an LC-MS system, facilitating its broad application in single-cell proteomics [Fig. 7C(ii)].

However, this technology still faces challenges. For instance, droplet uniformity, flow rate, and size distribution may affect chromatographic separation and MS signal intensity. Additionally, complex reaction conditions within the droplets can interfere with separation and detection results, requiring optimization of droplet generation and stability control to ensure data quality.

#### Other MS methods

In addition to the 3 common MS techniques, specialized methods can further enhance the efficiency and sensitivity of droplet analysis. Zhou et al. [91] developed an oil-free passive microfluidic system for directly injecting single cells into inductively coupled plasma quadrupole MS (ICP-QMS) [Fig. 7D(i)]. This system precisely introduces individual cells, allowing ICP-QMS to detect intracellular metal elements and reveal cellular heterogeneity. Combining different MS techniques can also leverage their respective strengths for higher sensitivity. Ha et al. [92] combined passive droplet loading with matrix-free laser desorption/ionization MS (NIMS), enabling direct deposition of enzymatic reaction droplets onto the NIMS surface for MS analysis without the need for additional sample handling (Fig. 7D(ii). D’Amico et al. [93] developed a droplet microfluidic platform integrated with ion mobility MS (IM-MS). By employing improved smoothing and interpolation algorithms, they reduced the data acquisition time 10-fold and successfully screened 96 compounds targeting the protein Sirtuin-5.

With the continuous advancement of MS, innovative methods have further improved the accuracy and sensitivity of droplet analysis. High-resolution MS enhances mass resolution and precision, enabling the detection of trace components in complex samples. When combined with chromatographic techniques like liquid or gas chromatography, these methods achieve better separation, boosting both sensitivity and specificity. Such integration not only improves droplet analysis resolution but also yields richer data in less time, accelerating applications in biomarker discovery, drug metabolism, and environmental monitoring. These developments are pushing MS toward more efficient, sensitive, and versatile use in life sciences and chemical analysis.

### Electrical detection

Electrical detection monitors changes in electrical signals, such as capacitance, resistance, and conductivity, to obtain real-time information about droplet size, velocity, and position. This method offers the advantages of noncontact, high sensitivity, and real-time monitoring, enabling precise tracking of the dynamic behavior of micro-scale droplets.

#### Impedance detection

**Fig. 8.**
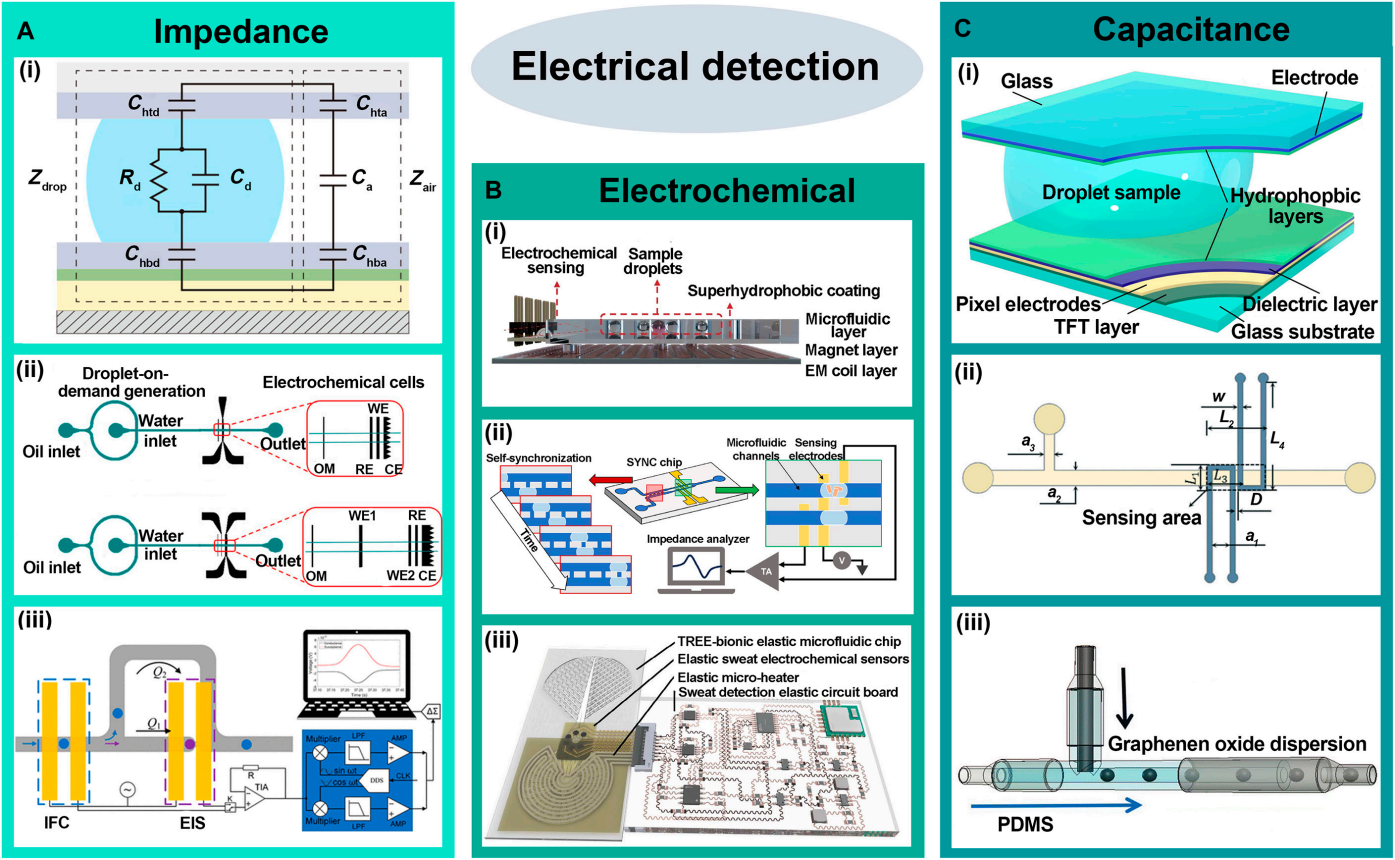
Electrical detection. (A) Impedance detection. (i) Impedance sensing system for digital microfluidics. Adapted from [94], Copyright 2024, Zeng et al., licensed under CC BY 4.0. (ii) Uses embedded electrode arrays for impedance detection. Reprinted with permission from [95], Copyright 2019, John Wiley and Sons. (iii) An IFC-EIS integrated microfluidic device. Reprinted with permission from [96], Copyright 2016, Royal Society of Chemistry. (B) Electrochemical. (i) PMDMF platform. Adapted from [98], Copyright 2025, Zhao et al., licensed under CC BY 4.0. (ii) Self-synchronized droplet-amplified electrical screening. Reprinted with permission from [99], Copyright 2024, Elsevier. (iii) Fully elastic wearable electrochemical sweat detection system. Reprinted with permission from [100], Copyright 2020, John Wiley and Sons. (C) Capacitance. (i) Capacitance-based droplet sensing system. Adapted from [101], Copyright 2024, Jiang et al., licensed under CC BY 4.0. (ii) Flexible microdroplet sensor with liquid metal electrodes. Reprinted with permission from [102], Copyright 2020, Royal Society of Chemistry. (iii) Flexible porous GO/PDMS capacitive pressure sensor. Reprinted with permission from [103], Copyright 2024, American Chemical Society.

Impedance detection enables high-sensitivity analysis of droplet size, composition, dynamic processes, and cellular states by monitoring the electrical characteristics of droplets, such as conductivity and dielectric constant. For example, Zeng et al. [94] proposed a new droplet impedance sensing system for digital microfluidics, as shown in Fig. 8A(i). This system can detect impedance over a wide frequency range and achieve the detection of droplet composition, size, and position. Additionally, Lombardo et al. [95] used embedded electrode arrays to measure electrical signal changes, combining impedance detection to precisely analyze droplet dynamics and chemical composition, as shown in Fig. 8A(ii). Furthermore, Feng et al. [96] developed a microfluidic device that integrates impedance flow cytometry (IFC) with electrical impedance spectroscopy (EIS), as shown in Fig. 8A(iii). The system passively captures single cells via hydrodynamic constriction and performs impedance measurements using coplanar electrodes. Zhong et al. [97] reported a system called DUPLETS (deformability-assisted dual-particle encapsulation via electrically activated sorting), which enables the screening of individual droplets by integrating mechanical and electrical properties, and actively selecting target droplets. The DUPLETS system embeds 2 pairs of microelectrodes in the microfluidic channel to characterize droplets based on electrical impedance and the transit time through a micro-constriction.

By monitoring the electrical characteristics of droplets, impedance detection can analyze droplet size, velocity, and composition in real time while capturing dynamic changes.

#### Electrochemical detection

Electrochemical detection is a technique that quantifies or identifies target analytes by measuring electrical signals generated from electrochemical reactions occurring at the electrode surface within droplets. Common detection methods include monitoring conductivity, current, potential, or electrochemiluminescence signals. This approach offers high sensitivity, rapid response, and ease of integration, making it especially suitable for real-time, label-free detection in droplet-based microfluidic systems.

Zhao et al. [98] developed a programmable magnetic digital microfluidic (PMDMF) platform integrated with an electrochemical detection system, as shown in Fig. 8b(i). By using a microcoil array and permanent magnets, the system enables noncontact, flexible droplet manipulation without high voltage or complex fabrication. Zhong et al. [99] proposed the self-synchronized droplet-amplified electrical screening cytometry (SYNC) system, which integrates droplet microfluidics with electrochemical amplification, as shown in Fig. 8b(ii). By encapsulating individual bacteria in picoliter-scale droplets and using a specially designed phosphate-amplified medium, the system converts bacterial metabolic activity into detectable conductivity changes, enabling real-time, fluorescence-free monitoring of bacterial growth. Niu et al. [100] proposed a fully elastic wearable electrochemical sweat detection system, as shown in Fig. 8b(iii). By employing a bionic tree-like structure, the system substantially enhances sweat collection efficiency, thereby enabling real-time multi-parameter detection using electrochemical sensors.

In summary, electrochemical detection provides a highly sensitive, rapid, and easily integrable approach for real-time, label-free analysis in droplet-based microfluidic systems. Its ability to measure conductivity, current, potential, or electrochemiluminescence within confined droplets enables precise analyte quantification.

#### Capacitance detection

Capacitive detection operates by measuring the capacitance changes resulting from differences in dielectric constants between droplets and the surrounding medium, providing dynamic data. For example, Jiang et al. [101] proposed a capacitive thin-film transistor-based digital microfluidic system featuring an inner–outer dual-pixel electrode structure that integrates both droplet actuation and sensing functionalities, as shown in Fig. 8C(i). In this design, discharge during droplet sensing occurs exclusively at the inner electrode, effectively avoiding droplet disturbance commonly caused by discharge in conventional sensing circuits. Zhang et al. [102] designed a flexible microdroplet sensor with liquid metal electrodes, leveraging the conductivity and flexibility of liquid metal to efficiently detect dynamic droplet changes, even for droplets of various shapes and sizes, as shown in Fig. 8C(ii). Similarly, Pan et al. [103] developed a high-performance flexible porous graphene oxide (GO)/PDMS capacitive pressure sensor using droplet microfluidic technology, as shown in Fig. 8C(iii). This method markedly improved pore size uniformity, thereby enhancing batch-to-batch consistency of the sensor.

In summary, capacitive detection technology offers substantial advantages in real-time monitoring and high sensitivity within droplet microfluidic systems. It can accurately capture droplet size, velocity, and position, facilitating droplet control and dynamic analysis. Although resolution in complex liquid systems still requires optimization, this technology is poised to play an increasingly crucial role in droplet microfluidics moving forward.

### TENG detection

The integration of TENG technology into droplet microfluidics offers distinct advantages. TENG converts droplet mechanical energy (e.g., movement or collision) into electrical signals, enabling high-sensitivity, noncontact, and self-powered detection. This allows real-time monitoring of droplet dynamics, including generation, movement, size, and velocity.

**Fig. 9.**
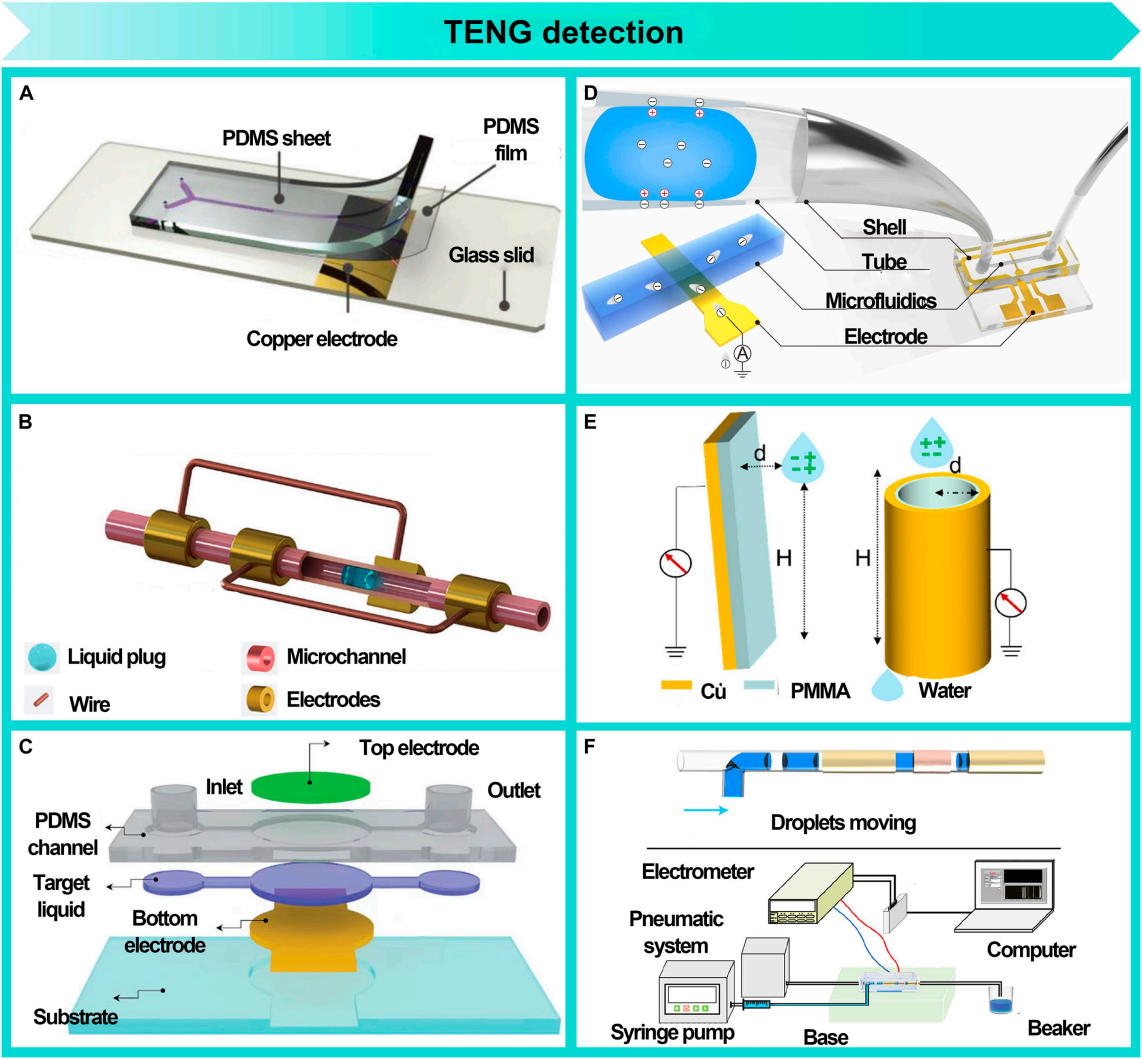
TENG detection. (A) Noninvasive self-powered droplet detection. Reprinted with permission from [104], Copyright 2023, John Wiley and Sons. (B) Grating electrode-based LS-TENG for noninvasive detection of droplet motion in opaque microchannels. Reprinted with permission from [105], Copyright 2024, John Wiley and Sons. (C) Single-electrode mode liquid sensor. Reprinted with permission from [106], Copyright 2018, Royal Society of Chemistry. (D) Triboelectric effect-based decoupled measurement method for droplet parameters in microfluidic chips. Reprinted with permission from [107], Copyright 2024, Elsevier. (E) Noncontact TENG based on electrostatic induction principles. Reprinted with permission from [108], Copyright 2023, Springer Nature. (F) Noninvasive droplet motion state monitoring method. Reprinted with permission from [109], Copyright 2021, American Chemical Society.

Liu et al. [104] demonstrated noninvasive, self-powered droplet detection by incorporating electrodes into a microfluidic chip (Fig. 9A). They monitored droplet generation frequency via voltage pulse frequency and tracked droplet length and velocity using a mathematical model. Zhao et al. [105] introduced a grating electrode-based liquid–solid TENG (LS-TENG), as shown in Fig. 9B. This technology enables real-time monitoring of droplet movement in the microchannel by generating electrical signals through the nanogenerator during the droplet’s motion. Kim et al. [106] presented a liquid sensor based on a single electrode mode utilizing the electrical reaction of the liquid with the external electric field generated by triboelectrification for liquid sensing (Fig. 9C). Hu et al. [107] proposed a decoupled measurement method based on the triboelectric effect for measuring droplet parameters in microfluidic chips, as shown in Fig. 9D. This method leverages the relationship between triboelectric signals and droplet characteristics, providing an efficient and noninvasive detection approach, capable of real-time monitoring of droplet behavior in complex environments. Deng et al. [108] developed a noncontact TENG based on electrostatic induction principles (Fig. 9E), effectively discerning droplet type, concentration, and frequency. Song proposed a noninvasive droplet motion state monitoring method (Fig. 9F), enabling stable monitoring of different liquid media, tilt angles, and droplet length through the correlation between voltage pulse count and liquid flow rate [109].

In summary, the integration of TENG into droplet microfluidics offers considerable advantages for noninvasive, real-time monitoring of droplet dynamics. Various studies have demonstrated the versatility of TENG-based systems in detecting key droplet parameters, including generation, size, velocity, and movement within microfluidic environments. These advancements highlight the wide-ranging potential of TENGs in applications such as single-cell analysis, drug screening, biomedical diagnostics, and intelligent monitoring systems. The high sensitivity, low cost, and ease of integration make TENG-based sensors a promising tool for a variety of fields, including medical diagnostics and industrial monitoring, offering a more efficient, cost-effective, and noninvasive alternative to traditional detection methods.

### Summary

Droplet detection plays a pivotal role in advancing the functionality and application scope of droplet-based microfluidic systems. Each detection method offers unique advantages: Optical techniques provide high-resolution, real-time visualization; MS enables precise molecular analysis; electrical detection allows label-free, integrable, and sensitive monitoring; and TENG-based detection introduces a novel, self-powered, and noninvasive approach. The integration of these techniques not only enhances detection accuracy and throughput but also broadens the applicability of microfluidics in fields such as biomedical diagnostics, environmental monitoring, drug discovery, and single-cell analysis. As microfluidic systems continue to evolve toward higher precision, miniaturization, and automation, the development of multi-modal, real-time, and portable droplet detection technologies will be essential to meet future research and industrial needs (Table 3).

**Table 3.** Summary of droplet detection methods

Category	Technology	Target	Advantage
Optical	Transmission and reflection	Droplet presence, size, velocity, and refractive index	High sensitivity, fast response, cost-effective
	Scattering and imaging	Droplet size, shape, merging, trajectory	Label-free, high resolution, dynamic analysis
	Fluorescence and spectroscopic	Biomarkers, concentration, reaction, pH, ions	High sensitivity, high selectivity, multiple parameter analysis
	Absorbance	Concentration, product, enzyme	Label-free, quantitative, high sensitivity
Mass spectrometric	ESI-MS	Proteins, nucleic acids, metabolites	High sensitivity, low fragmentation, gentle ionization
	MALDI-MS	Proteins, peptides, polysaccharides, polymers	Soft ionization, high sensitivity, rapid analysis
	LC-MS	Complex samples, multi-component	Excellent sensitivity and selectivity
Electrical	Impedance	Droplet presence, size, composition	Noninvasive, simple structure, low cost
	Capacitance	Droplet presence, size, composition	Noninvasive, real-time, low cost, simple structure
	Electrochemical	Biomarker, complex samples	High sensitivity, easy integration, trace analysis
TENG	TENG	Droplet dynamics, behaviors, size	Self-powered, low cost, easy integrated

## Droplet Microfluidic Applications

Droplet microfluidics technology holds considerable promise across various fields, particularly in single-cell analysis, 3D cell culture, drug development, and disease prevention. By precisely controlling the generation, movement, and distribution of droplets, this technology enables efficient single-cell-level analysis, providing insights into key biological processes such as cellular heterogeneity and gene expression. In 3D cell culture, droplet microfluidics offers an effective platform for simulating in vivo environments, facilitating the development of more physiologically relevant cell culture models. In drug development, it accelerates high-throughput screening and enables rapid reaction detection, expediting the discovery of new drugs. Moreover, in disease prevention, droplet microfluidics plays a crucial role in rapid diagnostics, disease biomarker detection, and the creation of personalized treatment strategies.

### Single-cell analysis

Droplet microfluidics, known for its efficiency and precision, has become widely used in single-cell analysis in recent years. This technology enables precise examination of cellular chemical composition, exploration of intracellular biomolecular information, and investigation of the physical properties of cells. The integration of these multidimensional data has made droplet microfluidics a vital tool for understanding cellular functions, uncovering cellular heterogeneity, and advancing research in disease and personalized medicine.

#### Chemical property

**Fig. 10.**
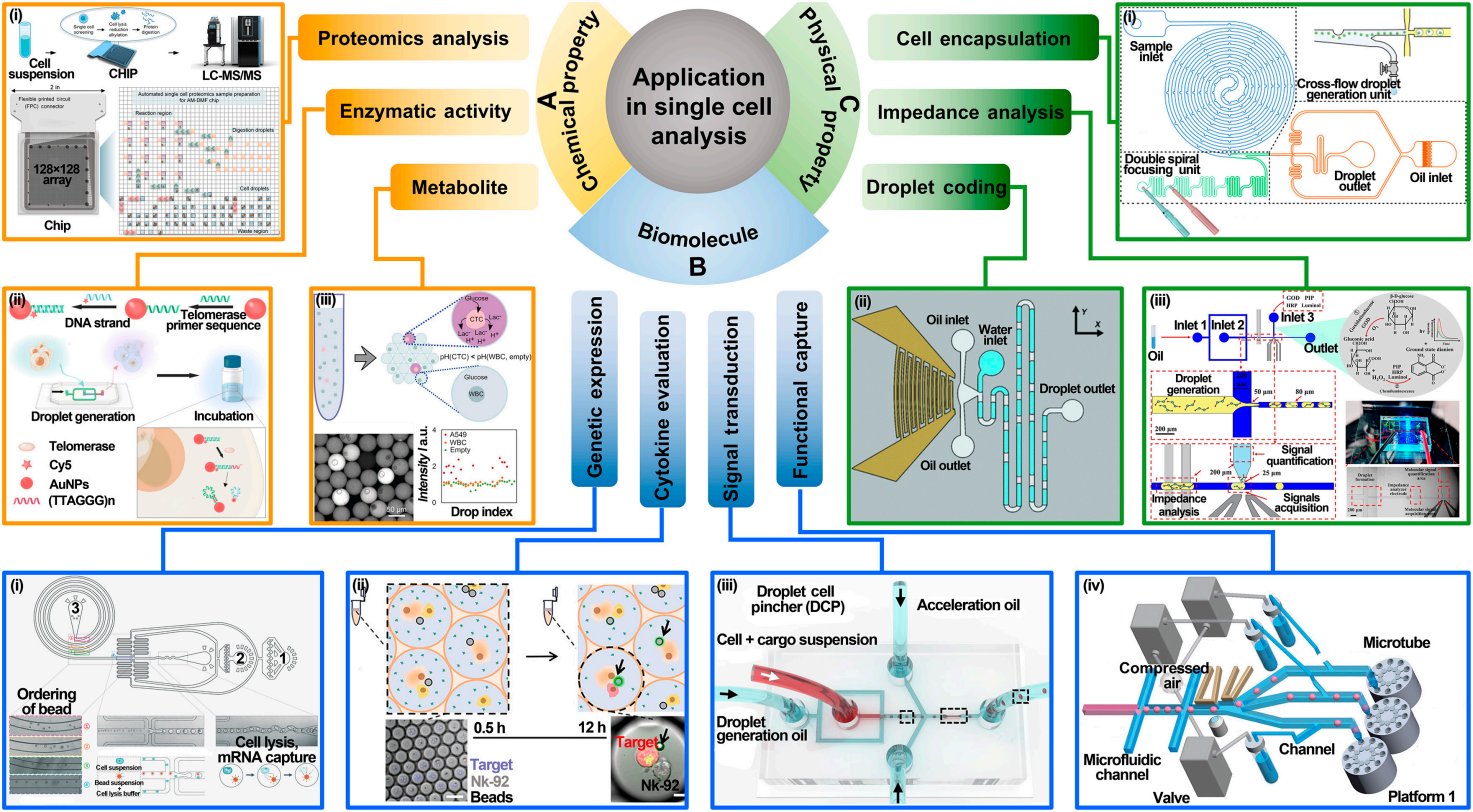
Application in single-cell analysis. (A) Chemical property. (i) Digital microfluidic chip with active matrix. Reprinted from [110], Copyright 2024, Yang et al., licensed under CC-BY-NC-ND 4.0. (ii) Droplet microfluidic platform using SERS and fluorescence dual-channel strategy. Reprinted with permission from [111], Copyright 2022, Royal Society of Chemistry. (iii) Method for detecting single-cell metabolites using droplet microfluidic platform. Reprinted with permission from [112], Copyright 2016, John Wiley and Sons. (B) Biomolecule. (i) High-throughput single-cell RNA sequencing via inertial sorting. Reprinted with permission from [113], Copyright 2018, Royal Society of Chemistry. (ii) Evaluating single-cell toxicity and cytokine release. Adapted from [114], Copyright 2020, Antona et al., licensed under CC BY 4.0. (iii) Microfluidic droplet-based CRISPR gene delivery platform with mechanical cell perforation. Adapted from [115], Copyright 2024, Kim et al., licensed under CC BY 4.0. (iv) Automated system for collecting defined droplets containing single cells. Reprinted with permission from [116], Copyright 2019, John Wiley and Sons. (C) Physical property. (i) Novel microfluidic chip for cell encapsulation. Adapted from [117], Copyright 2018, Wang et al., licensed under CC BY 4.0. (ii) Acoustic fluidics platform for droplet encoding. Reprinted with permission from [118], Copyright 2020, Royal Society of Chemistry. (iii) Method for determining liquid volume using impedance analysis. Reprinted with permission from [119], Copyright 2020, American Chemical Society.

Droplet microfluidics encapsulates individual cells within droplets, creating independent reaction environments for efficient and precise analysis of cellular chemical compositions, such as proteomics, enzyme activity, and metabolic profiling. Firstly, in proteomics, droplet microfluidics enables the analysis of protein composition and expression at the single-cell level. Yang et al. [110] combined active-matrix technology with digital microfluidics to create a large-scale, independently addressable electrode array using an active-matrix backplane. This innovation allows for high-throughput, parallel analyses, overcoming the limitations of traditional digital microfluidics due to restricted electrode size and number, as shown in Fig. 10A(i). Secondly, droplet microfluidics excels in measuring enzyme activity. Qi et al. [111] developed a droplet-based microfluidic platform using surface-enhanced Raman spectroscopy (SERS) and fluorescence dual-channel strategies for highly sensitive, in situ detection of low-abundance telomerase activity in single cells, as illustrated in Fig. 10A(ii). By introducing intelligent dual-responsive nanoprobe systems into the droplets, this method is capable of qualitatively and quantitatively measuring telomerase activity. Finally, in metabolic profiling, droplet microfluidics allows for precise detection of metabolic characteristics, such as the type and concentration of metabolites, to reveal the metabolic state of cells and their associations with diseases. Del Ben et al. [112] proposed a new method for detecting circulating neoplastic cells through single-cell metabolite analysis, shown in Fig. 10A(iii). By isolating single cells in microdroplets and analyzing their metabolic features, researchers can achieve high-throughput and accurate single-cell metabolic profiling.

#### Biomolecule

Droplet microfluidics has demonstrated unique advantages in exploring intracellular biomolecular information, including gene expression, cell activity factor release, signal transduction, and cell function assessment. In gene expression analysis, droplet microfluidics efficiently captures single cells for molecular-level transcriptomics. For instance, Moon et al. [113] developed an inertial sorting-based droplet microfluidics platform [Fig. 10B(i)]. The platform utilizes inertial effects in spiral channels for deterministic sorting of beads, ensuring precise encapsulation of individual beads and cells into droplets. For monitoring the release of cellular activity factors, droplet microfluidics provides isolated reaction environments to track secretion dynamics in real time. Antona et al. [114] developed a droplet-based microfluidic assay [Fig. 10B(ii)]. This method encapsulates natural killer (NK) cells, target cancer cells, polystyrene beads conjugated with interferon-γ (IFN-γ) capture antibodies, and fluorescent detection antibodies in water-in-oil (W/O) droplets. Fluorescent markers measure NK cell cytotoxicity and capture IFN-γ secretion around beads, revealing how increasing recombinant IFN-γ doses can adversely affect NK cell lytic activity. In signal transduction studies, droplet microfluidics precisely controls the microenvironment of single cells, such as adding specific stimulatory factors, to dynamically capture intracellular signaling pathway activation. Kim et al. [115] introduced a high-efficiency CRISPR gene delivery platform based on droplet microfluidic cell mechanical perforation [Fig. 10B(iii)]. The platform temporarily disrupts the cell membrane within droplets using a droplet cell pincher, enabling the efficient delivery of CRISPR-Cas9. Lastly, droplet microfluidics facilitates capturing and assessing overall cell functions**,** such as metabolic activity and motility. Nan et al. [116] proposed an automated system for accurately collecting droplets containing single cells [Fig. 10B(iv)]. The system alternately sorts and allocates droplets across 3 branched channels, precisely controlling droplet numbers. By integrating a nondestructive recovery strategy, this technology has achieved a 95% capture rate for individual cells.

#### Physical property

Droplet microfluidics has emerged as a powerful tool for analyzing the physical properties of individual cells. A considerable challenge in this technology has been balancing the cell suspension density with chip focusing performance during encapsulation. To address this, Tang et al. [117] proposed a novel microfluidic chip, as shown in Fig. 10C(i). The chip uses a double-helix structure to arrange cells nearly equidistantly, allowing the cells to be sequentially encapsulated in droplets. Droplet encoding plays a crucial role in large-scale single-cell analysis. Zhang et al. [118] developed an acoustic fluidics platform, as shown in Fig. 10C(ii). By using oil jets triggered by clustered traveling surface acoustic waves to dynamically separate water flow, droplets with determined volumes are continuously distributed at a rate of 100 Hz, with barcode information encoded through various droplet lengths. In impedance analysis, droplet microfluidics can rapidly analyze the physical properties of single cells, such as cell size, membrane capacitance, and conductivity differences in internal structures. For example, Yu et al. [119] employed impedance analysis to precisely measure droplet volume and used it as feedback to control the pressure for micro-sampling, ensuring that the sampling is unaffected by droplet volume, as shown in Fig. 10C(iii).

The application of droplet microfluidics in single-cell analysis not only provides high-precision methods for analyzing chemical compositions, intracellular biomolecules, and physical properties of cells but also advances precision medicine and personalized treatments. By continuously optimizing microfluidic chip designs and analytical techniques, droplet microfluidics enables high-throughput and accurate single-cell analysis, making it an indispensable tool in studies of cellular heterogeneity, oncology, immunology, and beyond.

### 3D cell culture

Building on single-cell analysis, droplet microfluidics enables precise control over microenvironments to create 3D cell culture models that simulate native tissue conditions. These 3D constructs are essential for studying cell behavior, differentiation, and drug responses under more realistic conditions than traditional 2D cultures.

Critically, 3D models integrate heterogeneity and functional data from single-cell studies, allowing for the construction of personalized tissue analogs, such as neoplasm organoids and fibrosis models. These biomimetic systems act as intermediary platforms linking cellular insights to practical applications like drug screening and disease modeling, creating a feedback loop that refines understanding and accelerates therapeutic development.

#### Physiological environment

**Fig. 11.**
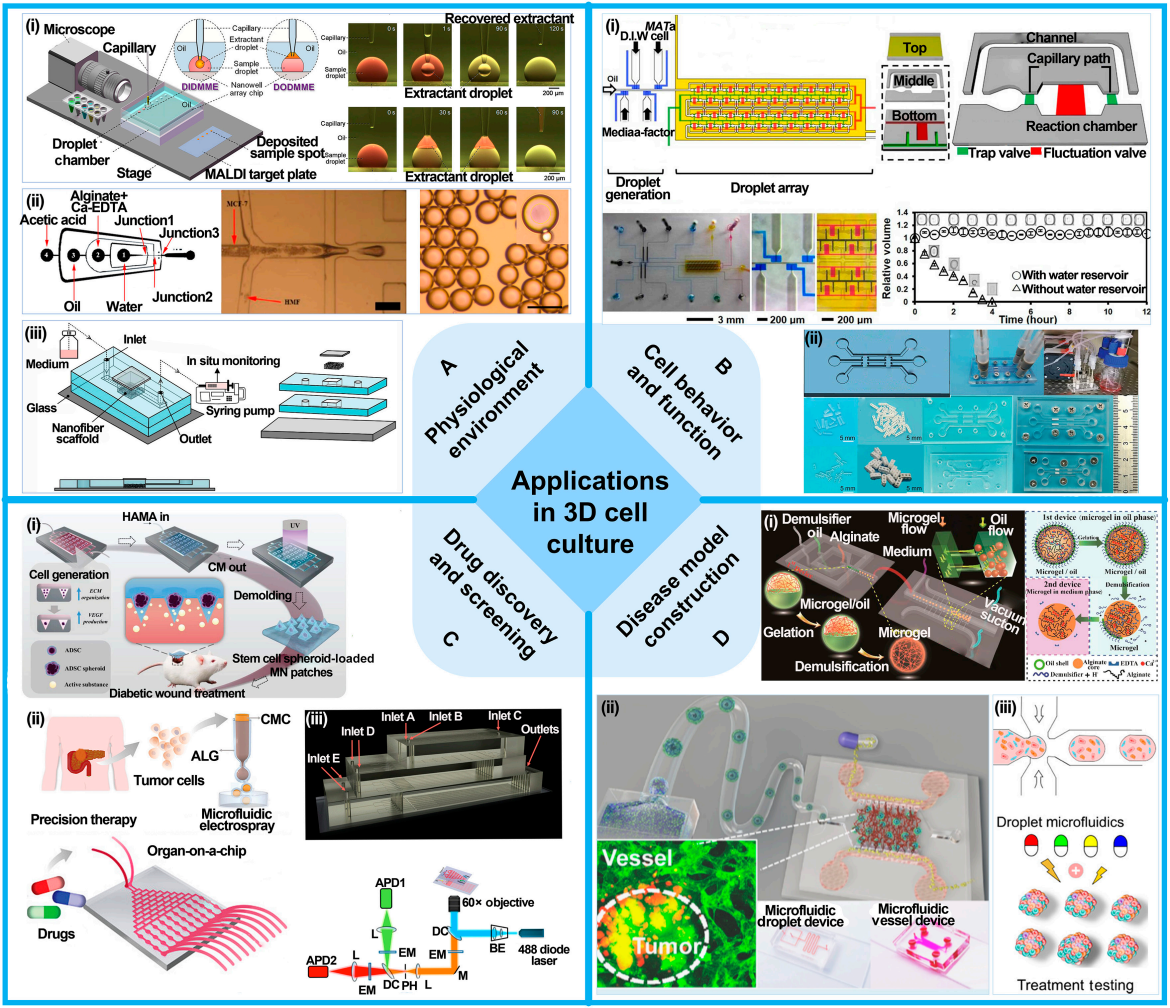
Applications in 3D cell culture. (A) Physiological environment. (i) Droplet–droplet microfluidic microextraction method. Reprinted with permission from [120], Copyright 2020, American Chemical Society. (ii) Microfluidic method for core–shell alginate particle production. Reprinted with permission from [121], Copyright 2018, American Chemical Society. (iii) Nanofiber scaffold-integrated microfluidic chip. Adapted from [122], Copyright 2018, Kim et al., licensed under CC BY 4.0. (B) Cell behavior and function. (i) Microfluidic platform for in situ detection of cell signaling. Reprinted with permission from [123], Copyright 2017, American Chemical Society. (ii) Microfluidic device with droplet interface double-layer array. Reprinted with permission from [124], Copyright 2020, John Wiley and Sons. (C) Drug discovery and screening. (i) Preparation of double-emulsion microcapsules using droplet microfluidic chip. Reprinted with permission from [125], Copyright 2023, John Wiley and Sons. (ii) Droplet microfluidic for high-throughput single-cell screening. Adapted from [126], Copyright 2023, Zhao et al., licensed under CC BY 4.0. (iii) Multilayer droplet microfluidic system. Reprinted with permission from [127], Copyright 2015, American Chemical Society. (D) Disease model construction. (i) Integrated microfluidic device for preparation cell-encapsulated microspheres. Reprinted with permission from [128], Copyright 2020, American Chemical Society. (ii) Integrated microfluidic system simulating vascular-supported neoplasm cell culture. Reprinted from [129], Copyright 2020, Wu et al., licensed under CC-BY-NC-ND 4.0. (iii) Method for generating 3D neoplasm spheroids. Reprinted with permission from [130], Copyright 2020, American Chemical Society.

Droplet microfluidic technology enables precise control over droplet generation and manipulation, thereby simulating complex physiological environments and promoting cell growth and differentiation during the culture process. Liquid extraction plays a crucial role in this process by enabling the selective removal and replacement of solutes within the droplets. Sun et al. [120] proposed a droplet–droplet microfluidic microextraction method that combines droplet microfluidics, liquid handling robots, and liquid-phase microextraction (LPME), as shown in Fig. 11A(i). Based on the solubility differences of extracting agents, 2 modes of microextraction are established: droplet-in-droplet and droplet-on-droplet, allowing micro-processing of ultra-small samples. Additionally, particle generation can provide unique advantages in simulating in vivo physiological environments. Droplet microfluidic devices generate uniform microdroplets, which, through further gelation, polymerization, or crosslinking reactions, can be transformed into particles with specific sizes and mechanical properties. These particles can serve as 3D culture matrices or scaffolds for cells, supporting cell attachment, growth, and differentiation. For example, Sun et al. [121] developed a microfluidic method for producing core–shell alginate particles, as shown in Fig. 11A(ii). Neoplasm cells are encapsulated in the core, and fibroblast cells are encapsulated in the shell, enabling the construction of complex neoplasm cell structures with good drug resistance. Furthermore, Kim et al. [122] proposed a microfluidic chip incorporating a nanofiber scaffold, as shown in Fig. 11A(iii). The electrospun nanofiber scaffold provides a 3D cell culture environment within the chip, and the perfusion method enables real-time monitoring of cell status based on conditioned media.

#### Cell behavior and function

Combined with microfluidic devices and real-time detection methods, the 3D environment encapsulated in droplets enables the real-time monitoring of single cells or cell populations, revealing dynamic changes and functional performance under physiological conditions. For example, Jin et al. [123] proposed a microfluidic platform for in situ detection of natural cell–cell contact and signaling processes within a closed microenvironment, as shown in Fig. 11B(i). The platform uses static droplet arrays, which efficiently capture, encapsulate, arrange, store, and culture specific cell populations for in situ monitoring of cell behavior. Moreover, droplet microfluidic technology has unique advantages in studying cellular functional characteristics, providing controllable microenvironments, high throughput, and dynamic monitoring. Ye et al. [124] integrated bioactive ceramics into a microfluidic chip to construct a ceramic micro-bridge microfluidic device, enabling the measurement of cell migration differences within the chip, as shown in Fig. 11B(i).

#### Drug discovery and screening

Droplet microfluidic technology allows for the simulation of in vivo microenvironments within miniaturized, controllable droplet systems, enabling dynamic testing of drug biological effects, screening of potential drug candidates, and optimization of drug delivery strategies. Microcapsule technology can encapsulate drugs in small oil–water emulsion droplets, precisely controlling droplet size and structure to ensure accurate drug delivery, enhance drug stability, and prevent premature release. Wu et al. [125] developed a novel stem cell spheroid-loaded microneedle patch using microfluidic chip technology, as shown in Fig. 11C(i). This system enables the efficient generation of uniform-sized stem cell spheroids and facilitates the precise delivery and exchange of multiple bioactive substances via microneedles, promoting tissue regeneration in diabetic wounds. Automated devices and microfluidic chips enable the simultaneous screening of large drug libraries and rapid assessment of their effects on 3D cells through integrated analysis systems. Song et al. [126] integrated a primary pancreatic cancer cell platform with a microfluidic chip and proposed a droplet-based microfluidic system for 3D tumor culture and clinical drug evaluation, as shown in Fig. 11C(ii). The system enables the rapid and stable formation of 3D tumor spheroids and facilitates dynamic, high-throughput assessment of chemotherapy regimens and drug effects. Kang et al. [127] proposed a multilayer droplet microfluidic system, as shown in Fig. 11C(iii), generating parallel droplet flows with varying concentrations. This system can perform different functions in each layer, such as parallel dilution, sample mixing, droplet generation, and long-term incubation.

#### Disease model construction

Droplet microfluidic technology, by precisely controlling the generation, movement, and merging of small droplets, enables the simulation of complex microenvironments within the human body, thereby promoting cell–cell interactions and tissue structure formation. Cellular microspheres can be used to simulate the growth, metastasis, and drug response of neoplasm cells, accurately replicating the heterogeneity of neoplasms and the complexity of the neoplasm microenvironment. By controlling the types and distribution of cells, the biological characteristics of neoplasm tissues can be more precisely modeled. For example, Zheng et al. [128] introduced an integrated microfluidic device for the preparation, online extraction, and dynamic culture of cell-laden microspheres, as shown in Fig. 11D(i). This method expands the noninvasive and nonsuppressive capabilities of droplet preparation systems, providing a stable microenvironment that reduces oxidative stress damage and mitochondrial accumulation within cells. Additionally, Wu et al. [129] proposed an integrated system to simulate a vascular-supported neoplasm for clinical drug screening, as shown in Fig. 11D(ii). This system uses droplet microfluidic technology to encapsulate Hct-116 neoplasm cells in microgel microspheres, simulating the interaction between neoplasm cells and extracellular matrix. Furthermore, Wu et al. [130] proposed a method for rapidly generating 3D neoplasm spheroids, as shown in Fig. 11D(iii). This method combines droplet microfluidics and scaffold materials to encapsulate neoplasm cells in oil-based matrix gel droplets, precisely controlling the number and composition of cells within the droplets. After removing the oil phase, the cells self-assemble under the support of the matrix gel, forming uniform neoplasm spheroids in a short period.

In conclusion, droplet microfluidic technology plays a significant role in 3D cell culture, enabling the precise simulation of the microenvironment for cell growth and supporting physiological processes such as cell proliferation, differentiation, and migration. This technology has also demonstrated broad prospects in disease model construction, drug screening, and drug delivery, providing an efficient, high-throughput research platform. Additionally, its innovative applications in cell function studies, drug effect evaluation, and neoplasm research are advancing the fields of regenerative medicine and personalized drug development.

### Drug development

Droplet microfluidics revolutionizes drug development by offering microscale synthesis, targeted delivery, and high-throughput screening capabilities. The technology’s capacity to produce uniform nanoparticles and microcapsules facilitates precise drug release control tailored to physiological cues such as pH and temperature.

Importantly, drug screening platforms based on droplet microfluidics leverage both single-cell data and 3D tissue models to evaluate efficacy and toxicity in conditions that closely resemble patient-specific microenvironments. This integrated approach enables rapid, cost-effective candidate validation, supporting the transition from bench to bedside with enhanced accuracy and personalization.

#### Drug synthesis

**Fig. 12.**
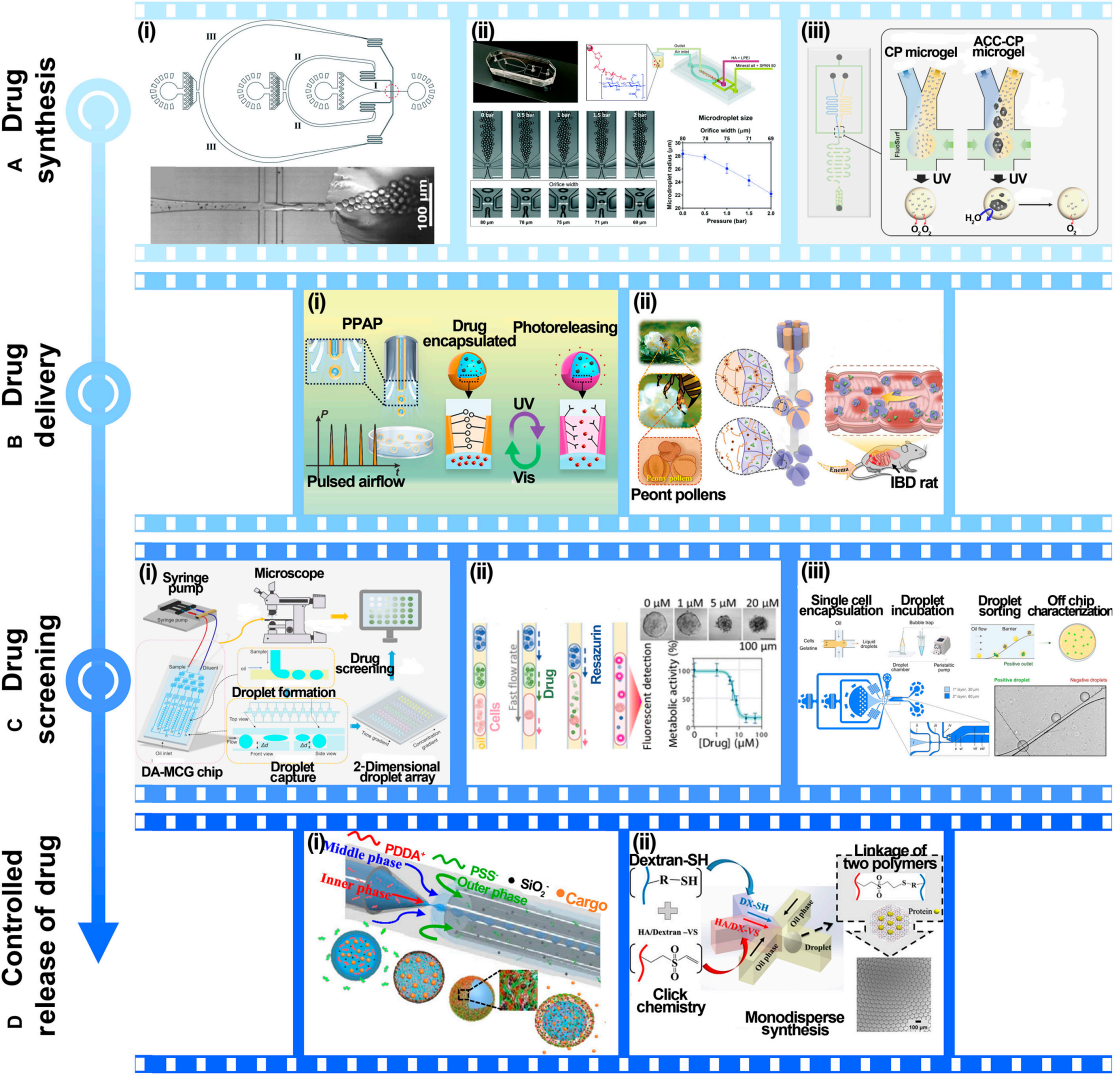
Application in drug development. (A) Drug synthesis. (i) Nanodrug hydrogel microcapsule based on Au@CoFeB-Rg3. Adapted from [131], Copyright 2021, Liu et al., licensed under CC BY 4.0. (ii) Pneumatic method for monodisperse nanoparticles. Adapted from [132], Copyright 2022, Giannitelli et al., licensed under CC BY-NC 3.0. (iii) Droplet microfluidics for oxygen-releasing dextran microgels. Adapted from [133], Copyright 20224, Tomioka et al., licensed under CC BY-NC 3.0. (B) Drug delivery. (i) Light-controlled microcapsules via PPAP. Reprinted with permission from [134], Copyright 2024, American Chemical Society. (ii) Multi-leaf-shaped microcapsule. Reprinted with permission from [135], Copyright 2023, John Wiley and Sons. (C) Drug screening. (i) Microfluidic chip for generating and capturing microdroplets. Reprinted with permission from [136], Copyright 2024, Elsevier. (ii) New drug screening platform. Reprinted with permission from [137], Copyright 2023, Royal Society of Chemistry. (iii) Separating protein-hydrolyzing microorganisms using passive droplets. Adapted from [138], Copyright 2024, Potenza et al., licensed under CC BY 4.0. (D) Controlled release of drug. (i) Microfluidic device for microcapsule formation. Reprinted with permission from [139], Copyright 2019, American Chemical Society. (ii) Drug encapsulation via click chemistry crosslinking technology. Reprinted with permission from [140], Copyright 2021, American Chemical Society.

Droplet microfluidics has shown tremendous potential in nanomaterial preparation, with its core advantage being the precise control over droplet size, internal reaction conditions, and the properties of the resulting materials. For example, Liu et al. [131] utilized this technology to develop an Au@CoFeB-Rg3-based nanodrug hydrogel microcapsule that enables precise drug release through external pH and temperature changes, showcasing its potential in intelligent drug delivery systems, as shown in Fig. 12A(i). Giannitelli et al. [132] further optimized the droplet microfluidic method, employing pneumatic driving for active adjustment of fluid dynamics focusing, which led to the preparation of highly monodispersed polymer nanoparticles, highlighting the technological advantage of droplet microfluidics in material uniformity and size control [Fig. 12A(ii)]. Tomioka et al. [133] developed uniform, monodisperse, and stable oxygen-releasing glucan microgels using photochemical crosslinking, as shown in Fig. 12A(iii). This approach provides a solution for hypoxic pathological environments and expands the application range of droplet microfluidics. These studies, ranging from drug delivery to material regulation and the development of novel functional materials, construct a logical chain centered around droplet microfluidics—from technological optimization to multifunctional applications—providing new research directions and application prospects in the fields of biomedicine and materials science.

#### Drug delivery

Droplet microfluidics technology enables the fabrication of various drug carriers, such as nanoparticles and microspheres, providing flexibility and tunability for targeted drug delivery. By controlling the composition and structure of droplets, multifunctional delivery systems can be designed to ensure that drugs are accurately delivered to the target sites. Guo et al. [134] utilized programmable pulsed air-dynamic printing (PPAP) technology to fabricate light-controlled microcapsules, achieving highly controlled drug release in terms of time, space, and dosage, as shown in Fig. 12B(i). Huang et al. [135] designed a novel multi-leaf-shaped microcapsule based on the natural form of peony pollen, as shown in Fig. 12B(ii). By increasing the contact area, they enhanced the targeted surface adhesion capability and combined it with a long-acting drug release system for effective local treatment of chronic inflammation. These 2 studies, focusing on dynamic matrix control and functional structure optimization, provide complementary approaches for developing the next generation of intelligent drug delivery platforms and offer more precise and efficient treatment options for complex and chronic diseases.

#### Drug screening

Microfluidic droplet technology enables high-throughput drug screening on a single platform. Each droplet can serve as an independent experimental unit for testing the activity, toxicity, and interactions of candidate compounds. Tan et al. [136] combined the generation of concentration gradients with droplet formation and capture, designing a microfluidic chip capable of dynamically generating and capturing microdroplets to form a static droplet array, as shown in Fig. 12C(i). By adjusting the aspect ratio of the horizontal channels and optimizing the oil-tree structure, they achieved uniform outlet flow while generating chemical concentration gradients, forming droplet arrays with varying concentrations. Parent et al. [137] proposed a new drug screening platform, shown in Fig. 12C(ii). Droplet microfluidics, triggered at programmable times, causes multiple droplets to merge, sequentially submitting spheroids for chemotherapy and cytotoxicity screening. Compared to microtitration methods, this system reduced the initial cell number by a factor of 10, opening new avenues for primary neoplasm drug screening methods. Potenza et al. [138] proposed a new passive droplet-based method for separating protein-hydrolyzing microorganisms, as shown in Fig. 12C(iii). Single cells were first encapsulated in gelatin microgel compartments and clonally cultured. High-throughput passive sorting of microcultures was achieved based on droplet deformability. These studies demonstrate the diverse applications of microfluidic droplet technology in drug screening, enhancing screening efficiency and providing a more precise and efficient platform for new drug discovery.

#### Controlled release of drug

The preparation of functionalized materials via droplet microfluidics enables the controlled release of drugs. The drug release rate can be precisely regulated based on the degradation characteristics of the material or external stimuli (such as pH, temperature, or light), thus improving therapeutic efficacy and reducing side effects. Zou et al. [139] proposed a microfluidic device for rapidly forming microcapsules, as shown in Fig. 12D(i). Using w/w droplets as templates, they successfully fabricated microcapsules that respond to external stimuli such as pH and osmotic pressure through polyelectrolyte complexation and the incorporation of silica nanoparticles. Chung et al. [140] employed a droplet-based microfluidic synthesis method, using click chemistry crosslinking to precisely fabricate hydrogel microspheres and encapsulate drugs, as shown in Fig. 12D(ii). The advantage of this method is its ability to achieve efficient and uniform drug encapsulation on a microfluidic platform, while controlling the drug release rate by adjusting the hydrogel’s crosslinking degree.

In summary, droplet microfluidic technology, as an advanced micro-nano fabrication and manipulation method, provides strong technical support for drug development, material preparation, and functional control. Particularly in the fields of intelligent drug delivery and precision therapy, droplet microfluidic technology not only enables timed and controlled drug release but also allows dynamic adjustments in response to external stimuli such as pH and temperature, thereby improving therapeutic efficacy and reducing side effects. In the future, with further technological advancements, droplet microfluidics is expected to open up broader prospects for personalized medicine, chronic disease treatment, and the discovery of new drugs.

### Disease prevention and treatment

In disease research, droplet microfluidics integrates insights from cellular analysis, 3D modeling, and drug testing to enable early diagnosis, therapeutic screening, and personalized treatment. Advanced disease models, such as neoplasm spheroids and patient-specific organoids, incorporate cellular heterogeneity and microenvironmental complexity, improving the fidelity of disease simulations. Furthermore, droplet microfluidic platforms for pathogen detection and immune cell screening harness precise droplet manipulation and molecular assays initially developed for cellular studies. This synergy accelerates the development of sensitive diagnostics and effective immunotherapies, positioning droplet microfluidics as a cornerstone technology in precision medicine.

#### Neoplasms

Cancer research has profound implications for improving human health. By investigating the mechanisms of neoplasm initiation, genetic mutations, and immune evasion, researchers can develop more effective strategies for early detection, diagnosis, and treatment. This not only accelerates the development of precision medicine but also lays the foundation for personalized cancer therapies.

**Fig. 13.**
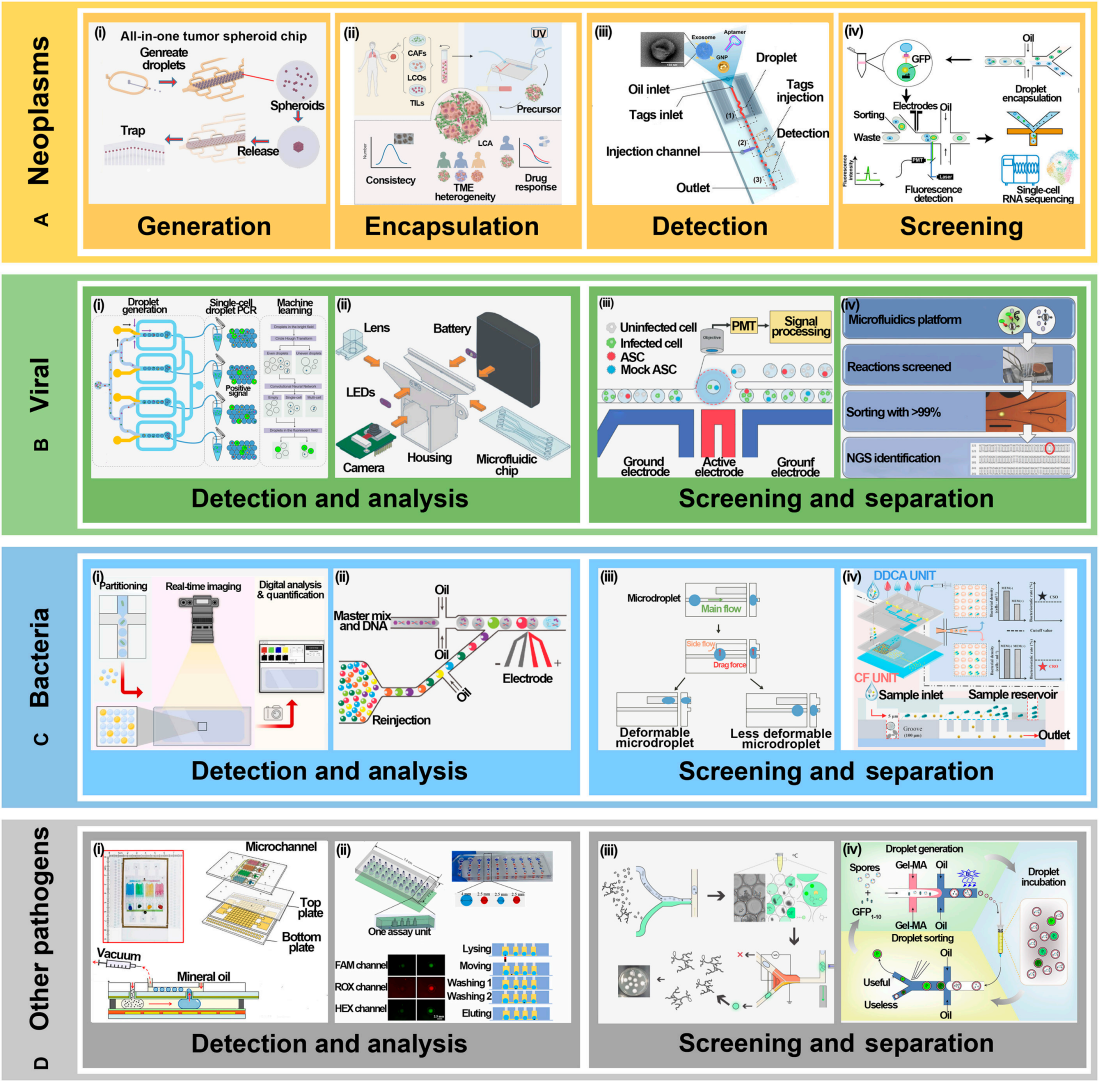
Application in disease prevention and treatment. (A) Neoplasm. (i) Scaffold-free 3D cellular spheroid generation. Adapted from [141], Copyright 2024, Petr et al., licensed under CC BY 4.0. (ii) Patient-specific LCA model. Adapted from [142], Copyright 2024, Wang et al., licensed under CC BY 4.0. (iii) Exosome detection in breast cancer. Reprinted with permission from [143], Copyright 2024, American Chemical Society. (iv) Tumor antigen-specific T cell screening and sorting. Reprinted with permission from [144], Copyright 2022, American Chemical Society. (B) Viral. (i) Multiplex droplet PCR for high-risk HPV detection in single cells. Reprinted with permission from [145], Copyright 2023, Elsevier. (ii) Bead detection system. Reprinted with permission from [146], Copyright 2020, Springer Nature. (iii) Screening virus-neutralizing antibody-secreting cells. Adapted from [147], Copyright 2022, Lin et al., licensed under CC BY-NC 3.0. (iv) Single-virus screening method. Reprinted with permission from [148], Copyright 2017, Elsevier. (C) Bacteria. (i) Live bacteria monitoring. Reprinted with permission from [149], Copyright 2024, Elsevier. (ii) CRISPR/Cas13a-based bacterial detection. Reprinted with permission from [150], Copyright 2024, Elsevier. (iii) Bacteria isolation via droplet microfluidics. Reprinted with permission from [151], Copyright 2023, American Chemical Society. (iv) Cascade filtration and droplet digital detection for bacteria. Reprinted with permission from [152], Copyright 2023, Elsevier. (D) Other pathogens. (i) Multiplex nucleic acid detection platform. Reprinted with permission from [153], Copyright 2024, American Chemical Society. (ii) Comprehensive pathogen detection chip. Reprinted with permission from [154], Copyright 2023, Elsevier. (iii) Filamentous fungi screening system based on enzyme activity. Adapted from [155], Copyright 2022, Samlali et al., licensed under CC BY 4.0. (iv) Screening system for filamentous fungi based on core–shell droplets. Reprinted with permission from [156], Copyright 2023, American Chemical Society.

The rise of neoplasm spheroids in cancer research is a direct result of their enhanced ability to simulate the in vivo neoplasm microenvironment, making them valuable for drug screening. However, challenges remain in achieving consistent spheroid formation and precise manipulation. Panuška et al. [141] introduced a novel droplet microfluidic method for generating uniform, scaffold-free 3D cell spheroids [Fig. 13A(i)]. This system streamlines the entire process, with all steps carried out within the microfluidic chip, eliminating the need for hydrogel encapsulation or external processing.

Precise manipulation and independent analysis of individual neoplasm cells within the microenvironment allow researchers to uncover the heterogeneity and complexity of neoplasms. Zhang et al. [142] developed an innovative patient-specific lung cancer-like glioma (LCA) model, shown in Fig. 13A(ii). Using droplet microfluidics based on microinjection strategies, they uniformly encapsulated patient-derived neoplasm microenvironment (TME) cells and lung cancer-like organoids within microgels, enabling more accurate modeling of patient-specific neoplasms.

Neoplasm detection is a key factor in cancer prevention and control, aiming to identify tumors early through efficient and precise techniques and improve the survival rate of patients. Ho et al. [143] developed a microfluidic platform for detecting HER2-positive exosomes in breast cancer cells, as shown in Fig. 13A(iii). The platform leverages salt-induced gold nanoparticle aggregation to amplify Raman signals, achieving a detection limit of 4.5 log_10_ particles/ml with a sample detection time of just 5 min.

Neoplasm antigen-specific T cell receptors (TCRs) are crucial in cancer immunotherapy as they can recognize and bind to specific neoplasm antigens, triggering an immune response that eliminates neoplasm cells. Wang et al. [144] developed a high-throughput microfluidic droplet platform for screening and sorting neoplasm antigen-specific T cells, as shown in Fig. 13A(iv). By encapsulating T cells in microdroplets and co-incubating them with antigen-presenting cells, this platform enables the efficient detection of T cell functional activity.

In summary, droplet microfluidics is a transformative tool that enables precise disease modeling, high-throughput screening, and personalized therapeutic strategies. By integrating droplet microfluidics with cutting-edge techniques like Raman spectroscopy and TCR screening, it holds great promise in revolutionizing early cancer detection and immunotherapy, and it is poised to become an integral part of the future of precision medicine.

#### Viral

Virus research is crucial to public health, disease prevention, and treatment. By gaining an in-depth understanding of the structure, mutations, and transmission mechanisms of viruses, scientists can develop vaccines, antiviral drugs, and immunotherapies to effectively address epidemics and cancer caused by viruses.

Droplet microfluidic technology considerably enhances the accuracy and speed of detection by dividing the sample into tiny droplets and using them as independent reaction chambers for virus capture and analysis. Huang et al. [145] proposed a multiplex droplet PCR method for detecting high-risk human papillomavirus (HPV) sequences in single cells, as shown in Fig. 13B(i). This method uses a multi-channel microfluidic chip with integrated 4 flow-focusing structures to prepare droplets. Through PCR, multiple target sequences are simultaneously detected based on a single fluorescence signal, and machine learning methods are used to analyze and identify a large number of single-cell droplets, achieving an accuracy rate of 97%. Additionally, Castro et al. [146] introduced a high-throughput bead detection system, as shown in Fig. 13B(ii). This system enhances detection sensitivity and throughput through a bead capture mechanism. Moreover, the Wi-Fi imaging module enables real-time remote monitoring, increasing convenience.

Droplet microfluidics technology not only enables rapid diagnosis even at low viral loads but also allows for in-depth studies of virus–host interactions, viral mutations, and more. Lin et al. [147] introduced a microfluidic platform for screening and enriching cells that secrete virus-neutralizing antibodies, as shown in Fig. 13B(iii). In addition, Chaipan et al. [148] introduced a high-throughput screening method based on single-virus droplet microfluidics, as shown in Fig. 13B(iv). This technique encapsulates individual virus particles in droplets and utilizes the microfluidic platform for precise manipulation of the virus and antibodies, enabling efficient screening of viral neutralizing epitopes.

#### Bacteria

Bacterial research is crucial to human health, environmental protection, and the development of biotechnology. By studying the biological characteristics of bacteria, their pathogenic mechanisms, and interactions with antibiotics, we can help develop new therapeutic approaches, especially in the context of the growing issue of antibiotic resistance. Furthermore, bacteria serve as fundamental tools in biotechnology, driving advancements in genetic engineering, vaccine development, and biopharmaceutical technologies.

Bacterial detection and analysis are critical aspects of microbiological research and clinical diagnostics, aiming to quickly and accurately identify bacterial species, quantities, and characteristics. Ki et al. [149] introduced a method for real-time monitoring of live bacteria, as shown in Fig. 13C(i). This system encapsulates bacteria in microdroplets using droplet microfluidics and, combined with wide-field imaging, allows for efficient, continuous observation and analysis of bacterial growth, division, and interactions with the external environment. Shang et al. [150] introduced a droplet microfluidic technology based on CRISPR/Cas13a for multiplex bacterial detection, as shown in Fig. 13C(ii). This method combines the CRISPR/Cas13a system with droplet microfluidics, enabling the simultaneous detection of multiple bacteria within a single reaction chamber.

Bacterial screening and separation are key steps in microbiological research, aiming to isolate specific bacterial species or phenotypes from complex samples. Muta et al. [151] introduced a microfluidic droplet screening method, as shown in Fig. 13C(iii). This method encapsulates bacteria with specific deformability in droplets and efficiently selects them based on their ability to degrade agar. Wang et al. [152] introduced a microfluidic detection method, as shown in Fig. 13C(iv). This method effectively isolates resistant microorganisms using cascade filtration, while droplet digital detection technology provides high-precision analysis of bacterial phenotypic characteristics.

#### Other pathogens

Research on other pathogens, including fungi, parasites, and prions, is also essential. These pathogens often cause severe yet frequently overlooked infections or diseases, such as invasive pulmonary infections caused by fungi, malaria, or schistosomiasis caused by parasites, and neurodegenerative diseases like Creutzfeldt–Jakob disease caused by prions. Studying these pathogens helps reveal their unique mechanisms of infection and transmission patterns, and facilitates the development of targeted diagnostic tools and treatments. Moreover, these pathogens are closely linked to the ecological environment, and their study provides a scientific basis for biodiversity conservation, agricultural disease control, and the risk assessment of cross-species transmission.

The detection and analysis of other pathogens plays a critical role in public health and clinical diagnostics. Traditional detection methods often have limitations, while emerging technologies such as droplet microfluidics, PCR, high-throughput genomics, and immunological detection offer efficient, sensitive, and cost-effective solutions. Xie et al. [153] introduced a multiplex nucleic acid detection platform [Fig. 13D(i)]. This method combines the flexible manipulation of digital microfluidics with the high-throughput characteristics of droplet microfluidics to achieve fully automated operations for sample preparation, nucleic acid amplification, and signal detection. Wu et al. [154] proposed a comprehensive detection platform based on a magnetic-controlled droplet microfluidic chip for rapid detection of pathogens and neoplasm mutation sites [Fig. 13D(ii)]. The chip uses precise manipulation of magnetic particles to integrate nucleic acid extraction, droplet distribution, and nucleic acid amplification into a closed system, achieving full-process automation.

Pathogen screening and sorting are critical steps in disease diagnosis and control. Samlali et al. [155] introduced a droplet digital microfluidic system for screening filamentous fungi [Fig. 13D(iii)]. The system uses droplets as independent micro-reactors, controlling the encapsulation of individual fungal spores within droplets while providing cultivation conditions and substrates to stimulate enzymatic reactions, enabling rapid assessment of enzymatic activity levels across different fungal strains. Zhang et al. [156] introduced a core–shell droplet-based microfluidic screening system [Fig. 13D(iv)]. The system encapsulates fungal spores in droplets with a core–shell structure, where biological or enzymatic reactions occur in the core region, while the shell layer provides protection against external interference. By precisely controlling the droplet size and reaction conditions, this system is capable of evaluating the specific enzyme activities, metabolites or growth characteristics of various fungal strains.

Droplet microfluidics provides an efficient, low-cost, and highly sensitive solution for disease research, with wide applications in the detection and analysis of neoplasms, viruses, bacteria, and other fields. In cancer research, droplet microfluidics helps generate and manipulate neoplasm spheroids to simulate the neoplasm microenvironment, advancing precision therapy. In virus detection, droplet microfluidics allows for high-throughput, rapid diagnosis even at low viral loads, providing strong support for vaccine development and antibody screening. In bacterial detection and screening, particularly in antibiotic resistance research, this technology demonstrates unique advantages in efficiently isolating and identifying bacterial species.

### Summary

The transformative potential of droplet microfluidics lies in its seamless integration across biomedical domains. By linking single-cell molecular characterizations with 3D tissue engineering, and further connecting these to advanced drug development and precise disease management, the technology forms a coherent, multidisciplinary research ecosystem.

Strengthening these cross-disciplinary connections is essential for maximizing the technology’s impact. Future research should prioritize enhancing data interoperability, standardizing platform interfaces, and fostering collaborative workflows that bridge gaps between fundamental biology, engineering, and clinical application. Such efforts will unlock broader prospects in personalized medicine, chronic disease treatment, and novel therapeutic discovery, fully realizing the value of droplet microfluidics within the biomedical industry chain.

## Challenges and Prospects

**Fig. 14.**
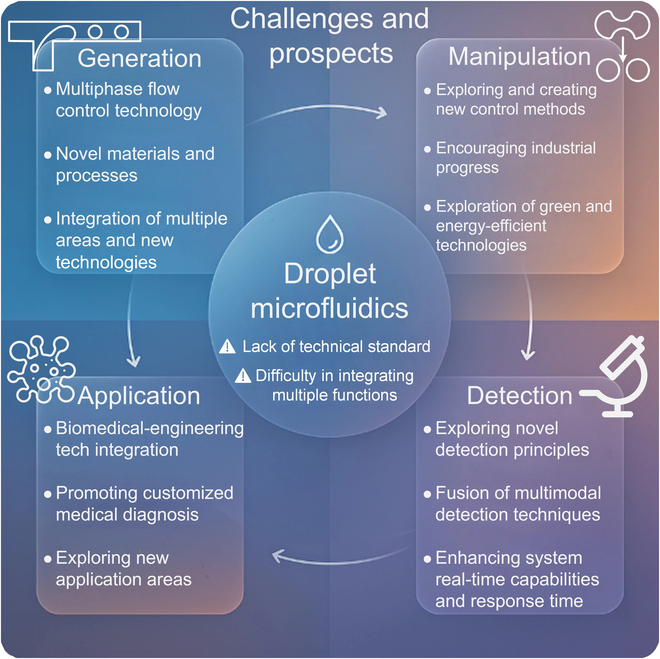
The challenges and prospects of droplet microfluidic technology.

Droplet microfluidics, as a cutting-edge research area in microfluidics, is rapidly advancing due to its extensive applications in high-throughput screening, single-cell analysis, and drug development. However, as the demand increases for multifunctional integration, operational precision, real-time monitoring, and high-sensitivity detection, the challenges faced by droplet microfluidics become more pronounced. This chapter will comprehensively explore the main technical bottlenecks of droplet microfluidics and anticipates future development trends and solutions in areas such as multifunctional integration, intelligent systems, and self-powering, thereby promoting the widespread application of droplet microfluidics in biomedical and chemical analysis fields. The challenges and prospects of droplet microfluidics are summarized in Fig. 14.

### Challenges

Current droplet manipulation within microfluidic chips remains limited by the constraints of channel structures, typically allowing only a single type of operation, such as droplet splitting or sorting in isolation. Few studies have successfully achieved multiple operations such as droplet mixing, splitting, sorting, and trapping simultaneously. To manipulate droplets with multiple operations, independent control of each droplet must be ensured while considering complex hydrodynamic interactions. Furthermore, realizing multiple operations requires complex structural design and operational stability, which pose substantial challenges. Therefore, a robust control and monitoring system is essential. The key to solving this issue likely lies in coupling various manipulation methods and detection techniques.

Droplet detection encounters 2 major challenges. First, in many biological analyses and environmental monitoring, the concentration of target substances is often low, and insufficient detection sensitivity may result in inaccurate identification and quantification. Concentrating or enriching the sample prior to detection can increase the concentration of target substances, or droplet manipulation within the chip can be employed to achieve this. However, pre-processing the sample before detection increases operational complexity and extends the detection cycle. While droplet manipulation within the chip offers a viable solution, integrating droplet generation, manipulation, and detection into a single system remains a substantial technical hurdle. Second, biochemical reactions within droplets are highly complex and necessitate dynamic monitoring. This is particularly crucial when reaction rates are rapid, as real-time monitoring is essential. Additionally, reactions within droplets often involve multiple substances and intricate reaction mechanisms, complicating the prediction of changes in reaction products. To address this, machine learning methods may be employed to analyze droplet reaction data in real time, identifying and predicting complex reaction patterns and products.

### Prospects

With continuous advancements in science and technology and increasing application demands, droplet microfluidic technology is gradually becoming an important tool in various fields, such as biomedicine, environmental monitoring, and material science. In the future, the development of droplet microfluidic technology will encounter numerous opportunities and challenges. Considerable progress is anticipated in the following areas: technology integration and optimization, self-power, intelligence and automation, and multifunctional application expansion. These developments will not only promote the commercialization of droplet microfluidic technology but also bring new possibilities for scientific research and industrial applications.

#### Multifunction integration

Multifunctional integration is a crucial development direction in droplet microfluidics. It aims to consolidate multiple functions—such as droplet generation, manipulation, detection, and reactions—into a single microfluidic system to enhance efficiency and flexibility. Integrating these functions can substantially reduce experimental steps and time, increasing overall efficiency, while allowing flexible adjustments to the experimental setup based on specific user needs. An integrated system also mitigates the risk of contamination during sample transfer and minimizes errors in sample handling while substantially lowering equipment costs. This is essential for the commercialization of microfluidic systems.

#### Self-powered system

Droplet microfluidic systems have vast application potential in outdoor environments, such as water quality testing, air quality monitoring, soil analysis, irrigation management, and mineral resource assessment. These environments frequently encounter the challenge of inadequate power sources. Therefore, self-powered droplet microfluidic systems represent one of the key future development directions in this field. The integration of TENGs with droplet microfluidics offers a promising solution to these challenges.

#### Intelligence and automation

Developing an automated control system based on machine learning and data analysis can facilitate real-time monitoring and regulation of droplet flow and reaction processes. By employing machine learning algorithms, the system can automatically learn and optimize droplet behaviors while analyzing the effects of different experimental conditions on reaction outcomes. This process not only enhances experimental precision but also reduces human error. This innovation will substantially improve the flexibility and reliability of experiments, marking a major advancement in the field.

#### Multifunctional application extension

The application of microfluidic technology in biomarker detection, drug screening, and cell analysis is poised for considerable growth, particularly to address the increasing demands for rapid detection and real-time monitoring. This growth will drive advancements in personalized and precision medicine. Additionally, microfluidic systems are expected to play a crucial role in the swift analysis of environmental samples, such as water quality testing and pollutant monitoring. By enhancing the efficiency of environmental protection and management, these systems will also help tackle the challenges posed by global environmental change.

In summary, the future of droplet microfluidic technology holds immense promise across various scientific and industrial fields. By advancing multifunctional integration, developing self-powered systems, and leveraging intelligence and automation, this technology will not only enhance the precision and efficiency of experiments but also broaden its application potential. As the demand for rapid, real-time, and personalized solutions grows, droplet microfluidics is poised to play a pivotal role in addressing complex challenges, fostering innovation, and contributing to sustainable development.

## Conclusions

Droplet microfluidic technology exhibits numerous advantages and exceptional performance across various fields. The latest advancements in droplet microfluidics are comprehensively reviewed, focusing on 4 key aspects: droplet generation, manipulation, detection, and applications. Detailed descriptions of micro-droplet generation techniques, encompassing both passive and active methods, are provided, along with a summary of the frequency and size range associated with different droplet generation approaches. Additionally, a range of operations such as droplet splitting, mixing, and screening within microchannels or planes is outlined. Furthermore, the potential of TENG technology as a novel power source for driving droplets on flat surfaces is evaluated, with an outlook on the promising synergies resulting from the integration of these 2 technologies. The review also consolidates the contributions of optical, electrical, and triboelectric methods in micro-droplet detection, highlighting the importance of micro-droplet parameter detection in practical applications of microfluidic technology. Finally, the diverse applications of droplet microfluidics in biology, medicine, and chemical analysis are summarized. This article summarizes and reviews the recent developments in the field of droplet microfluidics. As part of the accumulation of disciplines, it provides a reference for researchers in the field of droplet microfluidics and other disciplines that are expected to intersect with this field.
